# THOC1 complexes with SIN3A to regulate R-loops and promote glioblastoma progression

**DOI:** 10.1016/j.neo.2025.101271

**Published:** 2026-01-06

**Authors:** Shreya Budhiraja, Umme H. Faisal, Shivani Baisiwala, Rafal Chojak, Lara Koutah, Noah B. Drewes, Sia Cho, Hasaan A Kazi, Rebecca Chen, Ella N Perrault, Li Chen, Cheol H. Park, Maeve C. O’Shea, Khizar Nandoliya, Joseph T. Duffy, Peiyu Lin, Adam M Sonabend, Crismita C. Dmello, Atique U. Ahmed

**Affiliations:** a Department of Neurological Surgery, Feinberg School of Medicine, Northwestern University, USA; b Northwestern Medicine Malnati Brain Tumor Institute of the Lurie Comprehensive Cancer Center, Feinberg School of Medicine, Northwestern University, USA; c Northwestern University, Evanston, IL, 60208, USA

**Keywords:** Glioblastoma, THOC1, R-loops, Genome-wide CRISPR screen, Progression

## Abstract

Glioblastoma (GBM), the most common and aggressive primary malignant brain tumor in adults, has a median survival of 14.6 months. To identify drivers of GBM pathogenesis, we conducted a CRISPR-knockout screen, which revealed THO Complex 1 (THOC1) as a key driver. Knocking down THOC1 significantly reduced GBM cell viability across patient-derived xenograft (PDX) lines, enhancing survival (p<0.01) in primary PDX models. Conversely, overexpressing THOC1 in non-cancerous neural stem cells bolstered transformation capacity, decreasing survival and causing tumor engraftment *in vivo* (p<0.01). Further investigation revealed THOC1′s interaction with SIN3A, a histone deacetylase complex. Histone deacetylation has been previously shown to prevent the buildup of R-loops, structures that form normally during transcription but can be lethal in excess. We found that THOC1-knockdown leads to elevated R-loop levels and reduced histone deacetylation levels. RNA-sequencing analysis revealed that THOC1’s role in R-loop prevention primarily affects telomeres, critical regions for cell replication. We further show that THOC1-knockdown results in significantly increased telomeric R-loop levels and shortened telomeres. Ultimately, this study suggests that targeting THOC1 is a promising therapeutic strategy to disrupt the delicate R-loop landscape and undermine GBM's replicative potential.

**STATEMENT OF SIGNIFICANCE:**

Glioblastoma, the most aggressive malignant brain tumor in adults, relies on a delicate R-loop landscape to promote cell replication while avoiding DNA damage. Targeting THOC1 represents a promising therapeutic strategy to disrupt the delicate R-loop landscape and undermine GBM's replicative potential.

## Highlights

• CRISPR screen identifies THOC1 as a GBM progression driver
• THOC1 complexes with SIN3A to control R-loop homeostasis
• THOC1 loss increases R-loops and disrupts telomere stability
• Targeting THOC1 impairs GBM viability in PDX models
• THOC1 is a promising therapeutic vulnerability in GBM

## Introduction

A major hallmark of cancer is its ability to support the cellular machinery required for uncontrolled cell division [1]. One such mechanism is the resolution of R-loops which are RNA: DNA hybrids that form normally during transcription [2,3]. While R-loops play important roles in gene regulation, their unscheduled accumulation threatens genomic stability by inducing DNA double-stranded breaks [[2], [3], [4]]. In cancer cells, hyperactive transcription and replication increase R-loop formation, which can stall replication forks, causing replication stress and DNA damage [3]. Therefore, understanding how cancer cells have evolved to tackle R-loop accumulation may open new opportunities for therapeutic intervention.

Glioblastoma (GBM) is the most aggressive and common primary brain tumor in adults. Despite standard multimodal treatment involving surgical resection, radiation, and temozolomide (TMZ)-based chemotherapy, GBM has a median survival of approximately 14.6 months [5,6].

To identify the genes that promote GBM progression, our lab conducted a genome-wide CRISPR-Cas9 screen and found that THO Complex 1 (THOC1) is a significant contributor. THOC1 is a component of the transcription-export (TREX) complex, which links transcription to mRNA export by assembling on newly transcribed RNA. Previous studies have shown that loss of THOC1 impairs mRNA export and that THOC1 levels are elevated in several cancers, including prostate, ovarian, and colorectal [7]. However, its specific role in GBM remains unknown [[7], [8], [9]].

Our study demonstrates that THOC1 is a key regulator of GBM malignancy, exerting its effect through the control of R-loop homeostasis. Inhibiting THOC1 increases genotoxic R-loop accumulation, reducing tumor burden and extending survival *in vivo*. This R-loop-mediated mechanism offers new insights into how GBM cells maintain their replicative potential and evade DNA damage, positioning THOC1 as a promising therapeutic target.

## Methods and materials

### For detailed M&M, please see our supplementary data

#### CRISPR-Cas9 knockout screen

The Brunello library was transfected into H4 glioma cells (ATCC HTB-148 ™) which were subsequently treated with DMSO and harvested for sgRNA sequencing [10,11].

#### Cell lines and culture

Patient-derived xenograft (PDX) cell lines (GBM43, GBM6) were cultured in Dulbecco’s Modified Eagle’s Medium (DMEM) supplemented with 1% fetal bovine serum and 1% penicillin-streptomycin. Human glioma cell lines (U251, H4), neural stem cell, astrocyte, and fibroblast lines were cultured in DMEM/EMEM containing 10% FBS and 1% penicillin-streptomycin. NSC LM008 cells were cultured in neurobasal media supplemented with B27, N2, 1% penicillin-streptomycin, basic fibroblast growth factor (bFGF), and epidermal growth factor (EGF) [5,12].

#### Animals and *in vivo* models

Athymic nude mice were used in this study and housed according to Institutional Animal Care and Use Committee guidelines [13,14].

#### Cellular transfection and viral transduction

Lentiviral particles were generated using low-passage HEK293 cells according to a previous protocol [14]. The particles were then mixed with cells and spun for 2 h at 37°C at 850g [[14], [15], [16]].

#### Immunofluorescence and flow cytometry

Brain sections and cells were fixed, stained with antibodies, and analyzed [14,17].

#### Western blotting

Protein was extracted from cells, separated by SDS-PAGE, transferred to PVDF membrane, incubated with antibodies, and developed [18].

#### Dot blotting

Genomic DNA was extracted from cells, spotted onto nylon membranes which were incubated with antibodies and developed [19].

#### Immunoprecipitation

Protein samples were incubated with antibodies, then coupled with beads to isolate ubiquitin after a series of washes.

#### Quantitative PCR

RNA was extracted using Qiagen RNEasy kits and reverse transcribed using the iScript kit. qPCR was performed using a standard thermal cycler.

### Statistical analysis

Statistical calculations were conducted on GraphPad Prism v9.0. Additional experimental details are provided in the supplementary methods.

## Results

### Genome-wide CRISPR screen reveals THOC1 as a major driver of GBM aggression

**Fig. 1:**
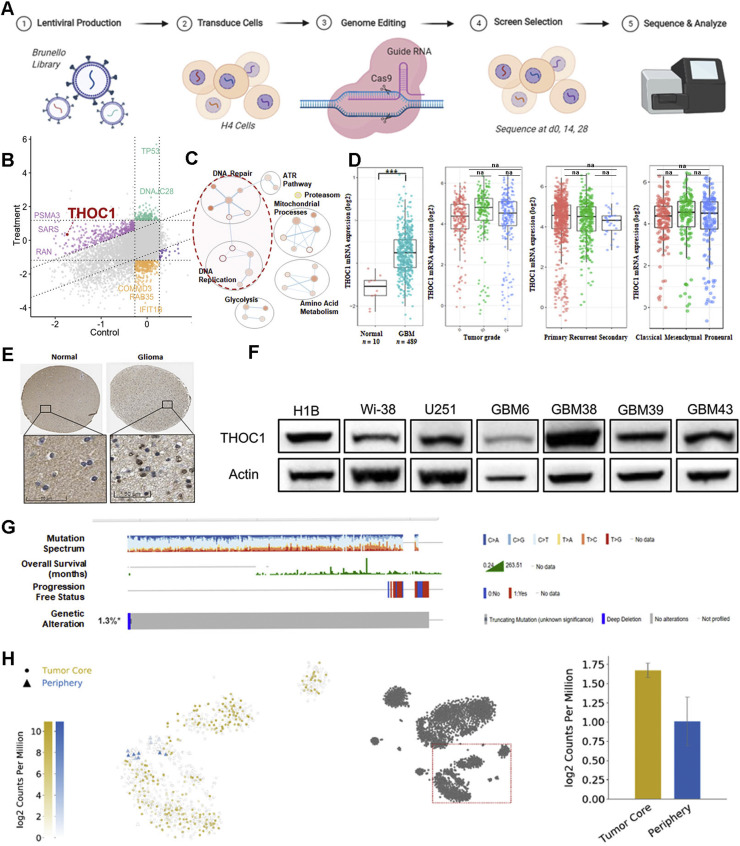
Genome-wide CRISPR Screen Reveals THOC1 as a Major Driver of GBM Aggression **A)** Schematic of the genome-wide CRISPR-Cas9 knockout screen in human H4 GBM cells using the Brunello library to identify genes critical for GBM progression. Sequencing at days 0, 14, and 28 highlights top depleted guides as potential oncogenic drivers. **B)** Identification of THOC1 as a significant hit from the CRISPR screen. **C)** Pathway enrichment analysis showing THOC1′s involvement in several key pathways identified in the screen. **D)** Elevated THOC1 mRNA expression in GBM compared to non-tumor samples, based on data from the GlioVis portal. **E)** Increased THOC1 protein expression in GBM tissue compared to normal brain tissue, as shown by Protein Atlas data. **F)** Western blot analysis showing higher baseline THOC1 expression in GBM cell-derived xenograft (CDX) line (U251) and patient-derived xenograft (PDX) line (GBM6, GBM38, GBM39, GBM43) compared to a non-cancerous line. **G)** cBioPortal data indicating a low mutation rate of THOC1 in GBM (approximately 5%). **H)** Single-cell RNA sequencing data from GBMSeq showing high THOC1 expression localized to the tumor core.

To identify genetic drivers of glioblastoma (GBM) progression, we performed a genome-wide CRISPR-Cas9 knockout screen in human H4 GBM cells using the Brunello library, covering over 19,000 genes with four sgRNAs per gene and 200+ non-targeting controls [20]. sgRNA abundance was assessed at days 0, 14, and 28. Depletion of specific guides over time was used to identify genes responsible for the oncogenic progression (Fig. 1A). Sequencing quality was confirmed through quality control metrics, including per-base sequence quality, per-sequence quality scores, and sequence length distribution (Fig. S1A). Gene read count distributions and sgRNA frequencies showed expected patterns across conditions, further confirming the reliability of the screen in identifying essential drivers of GBM through analysis of depleted guides (Fig. S1B). Finally, principal component analysis (PCA) assessment of similarities across replicates and differences across conditions substantiated screen quality (Fig. S1C).

Among the significantly depleted genes identified in our screen, THOC1 stood out due to its strong depletion score, reproducibility across replicates, and biological relevance (Figs. 1B, 1C, S1D). Pathway enrichment analysis (Fig. 1C) revealed that THOC1 is involved in multiple critical pathways associated with RNA processing, transcription termination, and DNA repair — hallmarks of GBM pathogenesis. While canonical essential genes such as TP53 and CDK1 also appeared as top hits, these represent well-characterized regulators, further adding to the robustness of the screen. In contrast, THOC1 has not been previously implicated in GBM and showed consistent dropout across guides, suggesting a novel role in maintaining glioma cell viability. Though SIN3A did not emerge as a top depleted gene in our CRISPR screen, we chose to investigate this gene due to prior evidence demonstrating interactions with THOC1 to prevent R-loop accumulation and preserve genomic stability [21].

To validate its relevance, we analyzed patient datasets. THOC1 mRNA expression – obtained from GlioVis portal patient data – was elevated in GBM samples compared to non-tumor tissue across multiple datasets [22] (Figs. 1D, S2A, S2B). Similarly, Protein Atlas revealed increased protein expression in GBM relative to normal brain tissue [23] (Fig. 1E, S2C). However, there was no significant correlation between THOC1 RNA expression and tumor grade, recurrence status, or tumor subtype (Figs. 1D, S2A, S2B). Western blot analysis further revealed elevated basal THOC1 expression across various GBM cell lines relative to a non-neoplastic control, with some variability among lines. (Fig. 1F). To account for potential subtype-specific effects, subsequent experiments were performed in multiple cell lines. Analysis of patient datasets from cBioPortal indicated a low THOC1 mutation frequency in GBM (∼5%) [24,25] (Fig. 1G). Single-cell RNA sequencing from GBMSeq showed that THOC1 expression is highly localized to the tumor core [26] (Fig. 1H) and elevated in contrast-enhancing regions relative to non-enhancing areas (Fig. S2D).

It is important to note that THOC1 expression was not influenced by temozolomide (TMZ), the standard of care chemotherapy for GBM. GlioVis patient data showed no increase in THOC1 RNA levels in patients treated with TMZ (Fig. S3A). Similarly, single-cell RNA sequencing of GBM cells treated with DMSO, TMZ, or collected mid-therapy revealed stable THOC1 expression, indicating that THOC1 expression is not altered during therapy (Figs. S3B, S3C). These results were corroborated at the protein level, as western blot analysis showed no trend or consistent change in THOC1 expression between DMSO- and TMZ-treated conditions at various time points in two cell lines, suggesting that THOC1 expression is not specific to TMZ therapy (Fig. S3D).

### THOC1 Shows Effects on GBM Viability In Vitro and In Vivo

**Fig. 2:**
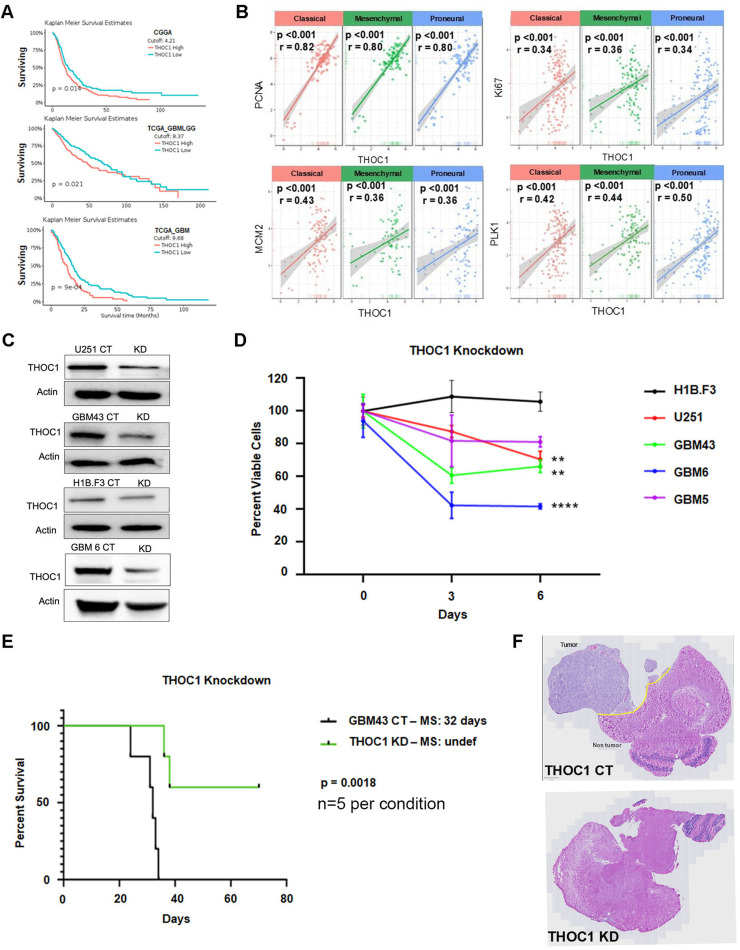
THOC1 expression is inversely correlated with survival **A)** Kaplan-Meier survival curves from GlioVis portal showing that higher THOC1 RNA expression correlates with shorter survival in GBM patients. **B)** Positive correlations between THOC1 RNA expression and proliferation markers (PCNA, Ki67, MCM2, PLK1). **C)** Western blot confirming effective THOC1 knockdown in GBM cell lines using CRISPR-Cas9, with substantial reduction in THOC1 protein levels. **D)** MTT assays demonstrating significant decreases in viability in THOC1-knockdown GBM cell lines, while neural stem cells (H1B.F3) remain unaffected. **E)** Kaplan-Meier survival analysis of mice implanted with THOC1-knockdown GBM43 cells, showing significantly extended median survival compared to controls. **F)** H&E staining of mouse brain tissue showing a marked reduction in tumor size in mice implanted with THOC1-knockdown GBM43 cells.

Survival analysis of publicly available GBM datasets (GlioVis) revealed that higher THOC1 RNA expression strongly correlates with shorter patient survival, corroborating its role in promoting GBM aggressiveness and clinical relevance as a potential therapeutic target (p<0.05) (Fig. 2A). To further validate this, we examined THOC1’s correlation with established proliferation markers *PCNA, Ki67, MCM2, and PLK1,* all of which showed positive correlation with THOC1 [[27], [28], [29]] (Fig. 2B).

To investigate functional relevance, THOC1 was knocked down via CRISPR-Cas9 constructs in H1B.F3, U251, GBM43, GBM5, and GBM6 cell lines (Fig. 2C). MTT assays revealed significant reductions in viability in GBM43, GBM6, and U251 (p<0.01), while neural stem cells (H1B.F3) showed minimal changes over the same period. (Figs. 2D, S4A). H1B.F3 cells are immortalized neural precursors that proliferate more slowly than malignant glioma cells and lack oncogenic transformation capacity; therefore, MTT signal increases only modestly over the first 6 days, reflecting limited growth kinetics [30,31]. The results of this cell viability assay were verified with additional sgRNAs, confirming reduced proliferation in THOC1-knockdown U251 and GBM43 cells relative to fibroblasts (p<0.05) (Fig. S4B).

In vivo, mice implanted with THOC1-knockdown GBM43 cells exhibited significantly increased median survival relative to control. While control mice had a median life of around 32 days, THOC1-knockdown mice had a significantly longer median survival (p<0.01, n=5 per group) (Fig. 2E). Post-mortem analysis confirmed THOC1 knockdown via IHC (Fig. S4C), and H&E staining showed that tumor size was reduced in mice implanted with THOC1-knockdown GBM43 cells (Fig. 2F).

### THOC1 overexpression results in tumorigenesis

**Fig. 3:**
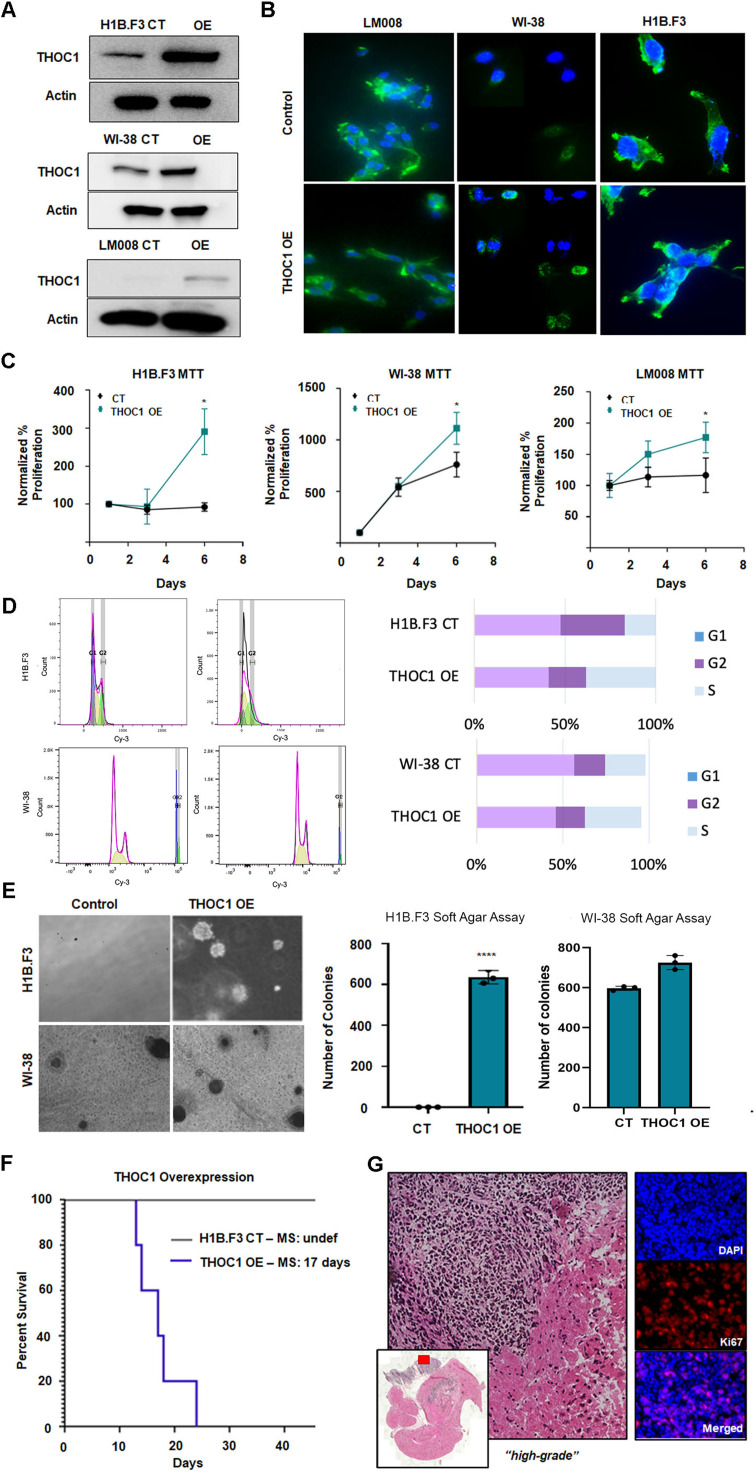
THOC1 expression promotes proliferation and migration **A)** Western blot confirming successful THOC1 overexpression in three non-cancerous cell lines (H1B.F3, NSC LM008, WI-38). **B)** Morphological analysis showing distinct process-like features in THOC1-overexpression cells, suggesting a more migratory, cancerous phenotype. Phalloidin (green) and DAPi (blue) stains were used to assess changes in morphology. **C)** MTT assays demonstrating significantly increased proliferation in THOC1-overexpression cells across all three cell lines, with H1B.F3 cells showing the greatest increase. **D)** Cell cycle flow cytometry analysis showing a higher percentage of THOC1-overexpression in H1B.F3 and WI-38 cells in the S-phase. **E)** Soft agar assay results showing large colony formation in THOC1-overexpression cells, particularly in H1B.F3, indicating transformation and anchorage-independent growth. Colony formation in WI-38 THOC1 overexpressed cells was increased compared to control cells, but not statistically significant. **F)** Kaplan-Meier survival analysis of mice injected with THOC1-overexpression H1B.F3 cells, showing accelerated decline and shorter survival compared to controls. **G)** Histopathological analysis of THOC1-overexpression tumors in mice, showing high-grade tumor development and significant Ki67 staining.

We then tested whether THOC1 overexpression could induce an oncogenic phenotype in normal neural stem cells. ORF constructs were used to overexpress THOC1 in H1B.F3 and LM008 (neural stem cell lines) and WI-38 (a fibroblast line) [32]. Western blotting confirmed successful overexpression (Fig. 3A), and morphological analysis of phalloidin, staining of F-actin revealed unique process-like extensions in THOC1-overexpression cells, suggestive of a more migratory character associated with this cancerous phenotype (Fig. 3B). In parallel, to assess whether this effect extended beyond neural stem cells, we generated a THOC1-OE in MO3.13 oligodendrocyte progenitor cells. Western blot confirmed increased THOC1 protein expression, which was accompanied by increased cell viability relative to control cells (Fig. S4D)

Tumor proliferation of THOC1-overexpression cells was measured via MTT assays and cell cycle flow cytometry. THOC1-overexpression cells in all three cell lines — H1B.F1, LM008, and WI-38 — showed significantly higher cell proliferation than control cells (p<0.05) (Fig. 3C), with H1B.F3 cells showing the greatest increase, reaching nearly 300% of control levels over six days (Fig. 3C). Additionally, cell cycle flow cytometry analysis showed a higher proportion of cells in the S-phase for both H1B.F1(38.39% vs. 17.07%) and WI-38 (32.80% vs. 23.40%) compared to respective controls. (Fig. 3D). We validated these findings using BrdU flow cytometry, which confirmed a significant increase in S-phase (BrdU⁺) cells in H1B.F3 following THOC1 overexpression, however, we did not notice the same change in WI-38 cell line. (Fig. S4F).

To examine the transformability in THOC1-overexpression cells, soft agar assays were performed. This assay evaluates a cell’s ability to grow in an anchorage-independent manner — a behaviour that is typically associated with cancer cells but not normal cells [33]. THOC1-overexpressing cells in both H1B.F3 and WI-38 lines formed large colonies. In H1B.F3 THOC1-overexpressing cells, approximately 600 colonies were observed, compared to none in the control (p<0.001) (Fig. 3E). In WI-38 THOC1 overexpressed cells, approximately 730 colonies were observed compared to approximately 600 in control cells. While there was an increase in THOC1 overexpressed cells in both cell lines, this change was only significant in the H1B.F3 cells.

We next assessed tumorigenecity of THOC1-overexpressing cells *in vivo*. H1B.F3 cells with control or THOC1-overexpression lentiviral vectors were injected into mice, which were monitored for tumor development and survival [34]. Brains were collected and sectioned for histopathology at endpoint. THOC1-overexpression resulted in accelerated decline and mortality when compared to controls, with THOC1-overexpression mice living an average of 17 days (Fig. 3F). Histological examination of harvested THOC1-overexpression tumors by an in-house licensed neuropathologist revealed tumor development, which was classified as “high grade.” Tumors also exhibited significant Ki67 staining, indicating highly proliferative nature [35] (Fig. 3G). Overall, these findings suggest that THOC1 overexpression has the capacity to induce a tumorigenic phenotype both *in vitro* and *in vivo*.

### THOC1 complexes with SIN3A to prevent R-loop formation in GBM

THOC1, or THO complex 1, canonically functions in the TREX (transcription/export) complex in order to regulate the export of polyadenylated RNA. However, recent studies have identified a novel interaction between THOC1 and SIN3A, a multi-protein complex that recruits HDACs 1 and 2 to facilitate transcriptional repression via histone deacetylation [21].

It has been widely shown that deacetylation is a prime mechanism for the prevention of R-loops, structures that form normally during transcription [36]. Specifically, by transiently closing the chromatin after passage of the RNA polymerase, HDAC recruitment renders the DNA inaccessible and unsuitable for forming R-loops. R-loops are ubiquitous three-stranded nucleic acid structures—consisting of a DNA:RNA hybrid that has displaced a ssDNA [37,38]. While R-loops can regulate gene expression, their accumulation threatens genomic stability by inducing DNA breaks and cell death [39].

**Fig. 4:**
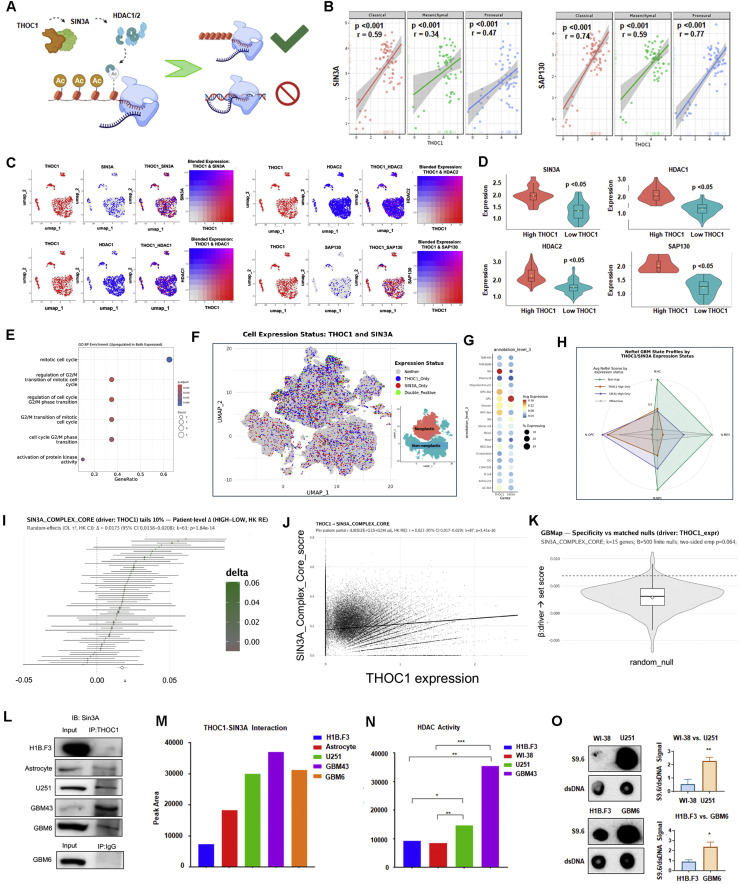
THOC1 Upregulation in GBM Enhances SIN3A-Mediated Deacetylation, Reducing R-loops and Promoting Cell Survival; Within-patient “KD-like” Contrasts Show Higher SIN3A–HDAC Core Activity in THOC1-HIGH Cells. **A)** Schematic representation illustrating the proposed mechanism by which increased interactions between THOC1 and SIN 3A in GBM promotes SIN3A-mediated deacetylation, thereby preventing R-loop accumulation and subsequent cell death. **B)** Correlation analysis of patient data from the GlioVis portal showing strong positive correlations between THOC1 and SIN3A, as well as SAP130 mRNA expression levels (p<0.001). **C)** UMAP FeaturePlots for single-cell sequencing of GBM43 cells implanted in vivo. Left: blended THOC1 (red) and SIN3A (blue); magenta marks cells with concordant high expression of both genes (blend.threshold=0.15). Grey indicates neither gene above threshold. Right: single-gene FeaturePlots for HDAC1, HDAC2, and SAP130 displayed on the same embedding. **D**) Stratification analysis from single-cell sequencing showing the correlation of THOC1 expression with SIN3A and associated genes at a single-cell level. **E)** Gene Ontology–Biological Process (GO BP) enrichment in THOC1+SIN3A+ cells, displaying the most significantly enriched terms. **F)** GBMap single-cell atlas highlighting THOC1+, SIN3A+, and THOC1+SIN3A+ populations, showing that double-positive cells are preferentially localized within malignant compartments. **G)** Dot plot summarizing THOC1 and SIN3A expression across GBMap cell types (dot size, fraction of expressing cells; color, average expression). **H)** Radar plot of Neftel lineage-state scores in THOC1+, SIN3A+, and THOC1+SIN3A+ cells. **I)** Random-effects meta-analysis (DerSimonian–Laird τ², Hartung–Knapp CIs) of per-patient contrasts between THOC1-HIGH and THOC1-LOW cells (10% tails; ≥20 cells/bin per patient) for the SIN3A complex–core score, where Δ = mean(module score in high-expressing THOC1 cells) − mean(module score in low-expressing THOC1 cells). Across patients, Δ = 0.0173 (95% CI as shown; k = 63; p = 1.84 × 10⁻¹⁴). **J)** Patient-level partial-correlation meta-analysis between THOC1 expression and SIN3A-core score, adjusted for library size, G1/S, and G2/M (Fisher-z, HK CIs); regression line and density contours shown for all cells. **K)** Specificity versus size/abundance-matched random gene-set nulls: target effect (dashed line) exceeds the null distribution (two-sided empirical p=0.064). **L)** Immunoprecipitation of THOC1 in GBM and non-cancerous cell lines, showing greater THOC1-SIN3A interaction in GBM lines compared to neural stem cells and astrocytes. **M)** Densitometry of immunoprecipitation demonstrating peak area**. N)** HDAC activity assays demonstrating higher HDAC activity in GBM cells compared to neural stem cells and fibroblasts (p<0.05). **O)** Dot blot analysis using the S9.6 antibody to detect DNA hybrids, showing greater R-loop levels in U251 and GBM6 cells compared to fibroblasts and H1B.3 neural stem cells.

Given their potential for genomic damage, R-loop homeostasis is critical. Dysregulated R-loops have been linked to several malignancies and may drive oncogenic progression [39,40]. Based on this, we hypothesized that THOC1 upregulation in GBM enhances SIN3A-mediated deacetylation required for preventing excess R-loop formation and thus cell death (Fig. 4A).

To investigate this, we first examined whether THOC1 expression correlates with SIN3A expression. GlioVis patient data indicated a strong correlation in mRNA expression between THOC1 and SIN3A, as well as SAP130, a known subunit of SIN3A (p < 0.001) (Fig. 4B). In vivo single-cell sequencing of GBM43 xenografts further confirmed that *THOC1*’s RNA expression is highly correlated *with SIN3A, SAP130, HDAC1, and HDAC2* at the single-cell level through stratification of low and high expression via optimal cutoff analyses (p<0.05) (Figs. 4C-4D). When analyzing the transcriptomic signature of *THOC1 and SIN3A* double-positive cells for pathway enrichment, the top pathways were associated with cell cycle-related gene signatures, including the mitotic cell cycle signature (Fig. 4E). Analysis of GBmap [41], publicly available single-cell RNA sequencing data from 338,564 cells of 110 GBM patients, revealed that *THOC1* expression is highest in Radial Glia (RG), while the coexpression of SIN3A and THOC1 is highest in the oligodendrocyte progenitor cell-like population (Figs. 4F-H).

Additionally, we found that THOC1 is associated with higher SIN3A–HDAC1/2 scaffold activity. In the canonical 10% tails contrast (≥20 cells/bin), THOC1-HIGH vs THOC1-LOW cells showed higher SIN3A complex–core scores (Δ=0.0173, k=63 patients, p=1.84 × 10⁻¹⁴) (Fig. 4I). A patient-level partial-correlation meta-analysis (adjusted for library size and proliferation) likewise supported a positive association (Fig. 4J). The observed effect exceeded the distribution of size- and abundance-matched random gene-set nulls (two-sided empirical p = 0.064), indicating moderate specificity of the SIN3A-core association (Fig. 4K).

CoREST and NuRD cores were likewise elevated in THOC1-HIGH cells, with effects attenuating in NON-OVERLAP variants lacking SIN3A-core components, supporting scaffold specificity. Consistent with this, SIN3A, but not SIN3B, showed the strongest modulation of HDAC-related gene signatures, and both THOC1 and SIN3A significantly influenced HDACi-responsive pathways (Figs. S5A-S5F). These single-cell transcriptomic analyses further substantiate the mechanistic model by revealing that THOC1 upregulation aligns with heightened SIN3A–HDAC1/2 scaffold activity across patient-derived GBM cells. This selective coupling to the SIN3A–HDAC axis, rather than generalized HDAC signaling, reinforces the notion that THOC1 maintains R-loop homeostasis through SIN3A-mediated chromatin regulation.

We next validated the interaction between THOC1 and SIN3A at the protein level. Immunoprecipitation of THOC1 in GBM and non-cancerous lines revealed stronger THOC1-SIN3A interaction in all three GBM lines compared to neural stem cells and astrocytes, supporting the notion that THOC1 upregulation in GBM may enable greater interaction with SIN3A (Fig. 4L, M). Previous reports have demonstrated that THOC1 and SIN3A induce the recruitment of major HDACs 1 and 2 which facilitate transcriptional repression via local histone deacetylation [21]. Next, to assess the HDAC activity in GBM cells, we performed an HDAC activity assay in which exogenous fluorophore-tagged peptides with acetyl groups fluoresced upon endogenous HDAC-mediated deacetylation [23]. GBM cells were found to exhibit more significant HDAC activity when compared to neural stem cells and fibroblasts (p<0.05) (Fig. 4N).

To determine if the R-loop levels are elevated in GBM, we conducted dot blot analysis using the S9.6 antibody, a highly specific antibody for DNA:RNA hybrids [42]. To ensure that S9.6 antibody specifically detects the R-loop but not the double-stranded RNA, all samples were treated with RNase H digestion [42,43]. Greater S9.6 signal was seen in U251 and GBM6 cells when compared to fibroblasts and H1B.3 neural stem cells, respectively, supporting the notion that GBM may require THOC1-mediated deacetylation to avoid toxic R-loop accumulation (p<0.05) (Fig. 4O).

### THOC1 mediates genomic stability through modulation of R-loops

**Fig. 5:**
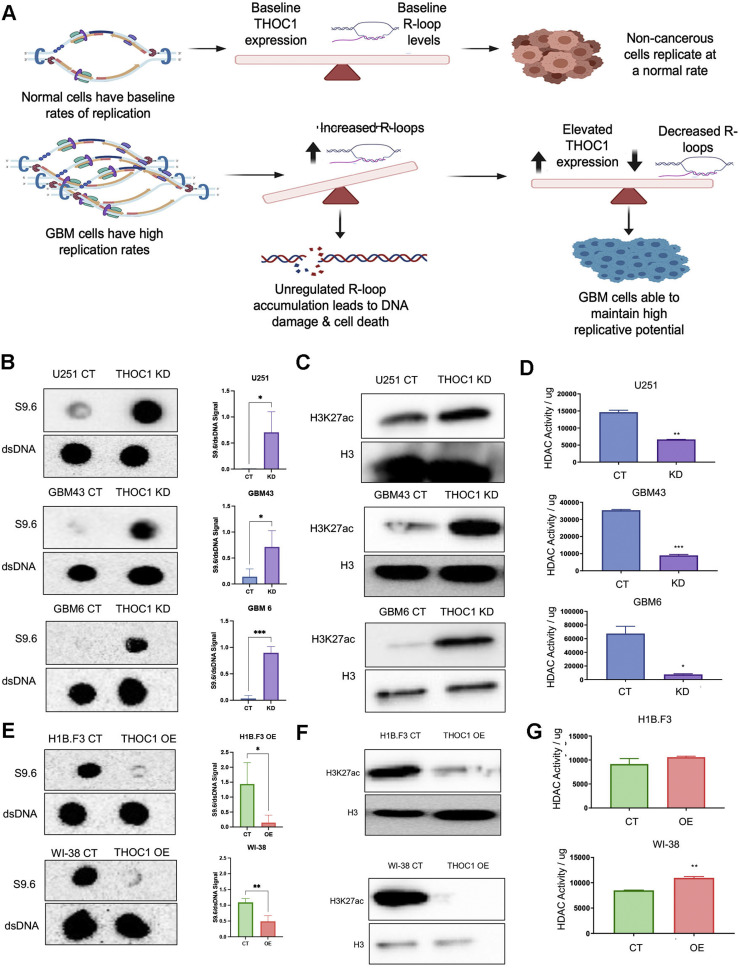
THOC1 promotes R-loops formation through modulation of HDAC activity **A)** Schematic representation of the hypothesized role of THOC1 in controlling R-loop levels in GBM cells to balance transcriptional activity and prevent DNA damage. **B)** Dot blot analysis showing a significant increase in R-loop levels in THOC1-knockdown GBM cells (U251, GBM43, GBM6) compared to control cells (p<0.01). **C)** Western blot analysis indicating increased global acetylation levels (H3K27ac) in THOC1-knockdown GBM cells. **D)** HDAC activity assays demonstrating significantly lower deacetylation activity in THOC1-knockdown GBM cells compared to control cells. **E)** Dot blot analysis showing that THOC1 overexpression in non-cancerous neural stem cells and fibroblasts significantly decreases R-loop levels. **F)** Western blot analysis showing lower acetylation levels (H3K27ac) in THOC1-overexpressing non-cancerous cell lines. **G)** HDAC activity assays showing increased HDAC activity in THOC1-overexpressing non-cancerous cell lines, with significant increases observed in WI-38 fibroblasts (p<0.05) and similar trends in H1B.F3 neural stem cells.

Non-cancerous cells may not exhibit elevated THOC1 expression due to their low basal replication and R-loop levels. In contrast, GBM cells may require THOC1 to counteract excessive R-loops. We hypothesized that THOC1 balances transcriptional R-loop formation to sustain GBM proliferation while preventing the harmful accumulation that would otherwise lead to DNA damage and cell death (Fig. 5A). We have previously demonstrated that THOC1 knockdown results in a decrease in viability in GBM cells. Based on our hypothesis, we postulate that in the absence of THOC1, GBM cells fail to handle the burden of high R-loop levels, leading to R-loop-associated DNA damage.

To examine this, we first measured S9.6 in control and THOC1-knockdown GBM cells. In all three GBM lines – U251, GBM43, and GBM6 – THOC1-knockdown resulted in a significant increase in R-loop levels, as compared to the control (p<0.01) (Fig. 5B). To identify whether this increased R-loop burden was due to THOC1’s association with SIN3A, we next assessed whether THOC1-knockdowns resulted in global changes in acetylation levels via western blot analysis. Indeed, greater levels of acetylation, as observed by H3K27ac, were observed in the knockdown conditions for all three cell lines – U251, GBM43, and GBM6 – confirming THOC1’s unique role in GBM’s to influence R-loop formation (Fig. 5C). HDAC activity assays similarly reflected the same phenomenon, with THOC1-knockdown GBM cells exhibiting significantly lower deacetylation activity (p<0.05) (Fig. 5D).

Given that THOC1 also demonstrated the ability to promote tumorigenesis when overexpressed in the primary cell line, we then sought to determine whether this THOC1 overexpression would result in R-loop biology similar to GBM cells. Dot blot analysis revealed that THOC1-overexpression significantly decreased R-loop levels in both normal neural stem cells and fibroblasts (p<0.05) (Fig. 5E). Furthermore, both lower acetylation levels and higher HDAC activity were concordantly observed in the THOC1-overexpression lines, as indicated by H3K27ac expression from western blot analysis and HDAC activity assays (Fig. 5F, 5G). Of the two lines, THOC1-expression in WI-38 fibroblasts demonstrated significantly increased HDAC activity (p<0.05), while THOC1-overexpression in H1B.F3 neural stem cells showed a similar trend (Fig. 5G).

### THOC1 enhances telomere stability through the prevention of telomeric R-loops

To better understand how THOC1 influences gene expression programs related to R-loop regulation in GBM cells, we performed bulk RNA sequencing of control and THOC1-knockdown cells, followed by gene set enrichment analysis (GSEA) to identify pathways affected by THOC1 loss.

**Fig. 6:**
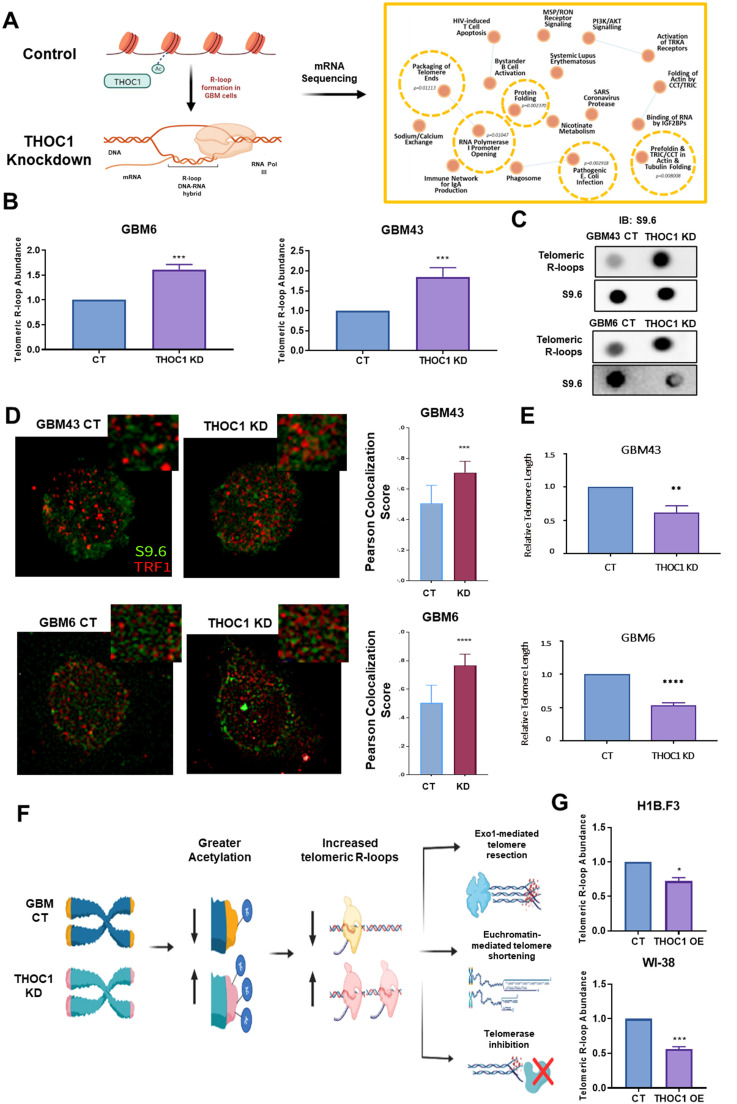
THOC1 enhances telomere stability through prevention of telomeric R-loops **A)** GSEA analysis of bulk RNA-sequencing data from THOC1-knockdown and control GBM cells, revealing downregulated pathways including telomeric packaging, protein folding, and RNA polymerase I promoter opening in knockdown cells. **B)** Comparative analysis showing a significant increase in telomeric R-loop abundance in THOC1-knockdown GBM cells. **C)** Dot blot and immunoprecipitation analysis with S9.6 antibody, showing increased telomeric R-loops in THOC1-knockdown cells compared to control cells. **D)** Immunofluorescence staining of telomere marker (TRF1) and R-loops (S9.6) showing greater co-localization of TRF1 and S9.6 in THOC1-knockdown GBM cells compared to control cells, indicating elevated telomeric R-loop presence. **E)** Analysis of telomere length in THOC1-knockdown cells showing significantly reduced telomere lengths. **F)** Schematic model proposing that THOC1’s influence on GBM viability and progression may be predominantly driven by its role in suppressing telomeric R-loops rather than global R-loop levels. **G)** Analysis showing decreased telomeric R-loop abundance in THOC1-overexpressing neural stem cells and fibroblasts.

Analysis of pathways most downregulated in THOC1-knockdown cells revealed telomeric packaging, protein folding, and RNA polymerase I promoter opening as the most enriched pathways (Fig. 6A). telomeric repeat-containing RNA (TERRA), transcribed from subtelomeric regions, base-pairs with telomeric DNA to create R-loops [3]. Transient telomeric R-loops- may help sister telomers during cell division [44]. Telomeric R-loops are double-edged swords as they can support telomeric maintenance, but they must be tightly controlled to prevent genomic instability.

To further understand if THOC1 may influence telomere homeostasis via R-loops in GBM, we compared telomeric R-loop abundance in control and THOC1-knockdown cells. We observed a significant increase in telomeric abundance via qPCR in THOC1-knockdown GBM cells (Fig. 6B). We further validated these results via dot blot analysis of telomeric isolates, followed by immunoprecipitation of S9.6, in the same cell lines, which exhibited an increase in telomeric R-loops in THOC1-knockdown cells relative to the controls (Fig. 6C). This was also seen via immunofluorescence staining of telomere marker (TRF1) in red and R-loops (S9.6) in green, which showed significantly greater co-localization of TRF1 and S9.6 in THOC1-knockdown cells compared to control cells (Fig. 6D).

Given this increased presence of telomeric R-loops in THOC1-knockdown cells and their implications on telomere damage, we next investigated whether increased abundance of these structures resulted in reduced telomere length, a proxy for overall telomeric health. Indeed, we observed that THOC1-knockdown cells exhibited significantly reduced telomere lengths, suggesting that THOC1 may play an even more crucial role in regulating the global R-loop landscape: specifically suppressing R-loops on telomeres to ensure telomeric maintenance (Fig. 6E).

We have previously shown that THOC1’s influence on R-loops in GBM cells is the result of its role in the epigenetic process of deacetylation, which may lead to increased telomeric R-loops as well. However, given the unique implications of R-loops specifically on telomeres – including the promotion of Exo1-mediated resection, euchromatin-mediated telomere shortening, and telomerase inhibition – perhaps the effect of THOC1 on GBM viability and progression that we see is predominately driven by its role in influencing telomeric R-loops specifically, rather than global R-loop levels (Fig. 6F). Additional studies revealed that THOC1-overexpression in neural stem cells and fibroblasts result in significantly reduced telomeric R-loop abundance and increased telomeric length in both cell lines, highlighting the possibility that similar mechanisms may be at play in THOC1’s role in tumorigenesis (Fig. 6G, Fig. S6A). The relationship between R-loop accumulation and genomic stress is still under investigation [45]. In our initial experiment, we indicate that increased levels of R-loops lead to increased DNA damage (Fig. S6B).

To investigate THOC1 as a therapeutic target, we identified luteolin, an inhibitor of THOC1 that has shown efficacy in the proliferation of other malignancies [46]. However, luteolin has not been studied in the context of GBM and was seen as a favorable agent due to its blood-brain barrier permeability [47]. We tested GBM viability after treatment with 50 uM luteolin, which decreased cell proliferation to approximately 60% (Fig S7A). To ascertain whether luteolin-induced THOC1-knockdown also resulted in greater levels of R-loops, acetylation, and DNA damage, immunocytochemistry staining in U251 and GBM43 cells comparing non-treated cells to 50 μM luteolin-treated cells revealed not only higher levels of S9.6 fluorescence but also higher levels of DNA damage and acetylation (Fig S7B). Dot blot analysis similarly revealed significantly higher S9.6 signal in luteolin-treated cells (p<0.05) (Fig S7C). To determine if this mechanistic change induced by luteolin is translated *in vivo*, we implanted GBM6 cells into mice intracranially and treated with DMSO or luteolin for 5 days. After death from tumor burden, both tumor and non-tumor cells were harvested in both treatment conditions. R-loop levels were measured using dot blot analysis (Fig S7D). Luteolin-treated tumors displayed substantially greater S9.6 signal than DMSO-treated tumors, indicating that luteolin does indeed induce THOC1-knockdown to allow for R-loop accumulation (p<0.01). Normal brain did not reveal any differences in R-loop levels in DMSO-treated cells and luteolin-treated cells, suggesting that luteolin is not toxic and only has an effect on GBM cells (Fig S7E). We also evaluated cell viability with the current standard of care for GBM, temozolomide, and found that higher concentrations of TMZ lead to decreased cell viability as luteolin dose increases, suggesting an additive effect in U251, GBM6, and GBM43 (Fig. S7F).

## Discussion

One of the major hallmarks of GBM, like many other cancers, is its ability to thrive despite genomic instability. This instability drives the stepwise accumulation of mutations that contribute to its heterogeneous and aggressive phenotype [48,49]. Within this context, R-loop-induced genomic instability emerges as a significant factor in the progression and oncogenesis of GBM. However, the role of R-loops in GBM remains unclear due to their paradoxical nature. On the one hand, they are critical for meeting the transcriptional demands of uncontrolled cell division. In fact, R-loops serve as essential intermediaries in gene expression, playing a role in regulating various cellular processes. On the other hand, excessive or dysregulated R-loop formation can compromise genomic stability, resulting in cell death [50]. Given this dual role, our study aimed to elucidate how GBM straddles this fine line between R-loop deficiency and excess to promote GBM progression.

THOC1 is elevated in various cancers including breast, hepatocellular, and colorectal cancer where it appears to be essential for sustaining neoplastic transformation, as tumor cells depend on its function to maintain malignant growth [46,51]. In breast cancer, THOC1 expression was elevated compared with normal epithelium, and elevated levels correlate positively with tumor size and metastatic progression [52]. Despite this emerging evidence across tumor types, the role of THOC1 in glioblastoma remains poorly defined.

We first showed that THOC1 is elevated in GBM compared to non-cancerous cell lines. Knocking down THOC1 significantly decreased GBM viability when compared to non-cancerous cell lines, suggesting its unique role in GBM progression. *In vivo* studies using a GBM43 PDX model revealed that mice implanted with THOC1-knockdown cells had a significantly increased median survival and reduced tumor size, emphasizing THOC1′s role in tumor growth and aggressiveness. We then assessed the effects of THOC1 overexpression in non-cancerous lines. Our *in vitro* studies reveal that THOC1 overexpression results in increased cell proliferation, cell cycle progression, and anchorage-independent growth, highlighting THOC1’s ability to promote a tumorigenic phenotype. In our study, THOC1 overexpression promoted tumorigenesis in fibroblasts, demonstrating tumorigenic capacity outside of neural contexts. THOC1 overexpression also led to tumorigenic properties in neural stem cells, which are related to glioblastoma. Future studies in glioma-prone cells will further clarify the role of THOC1 in promoting tumorigenesis. Moreover, *in vivo* experiments demonstrated that THOC1-overexpressing non-cancerous cells were capable of inducing tumor engraftment in mice, indicating the oncogenic potential of THOC1 and its role in transforming normal cells into a cancerous phenotype.

To explore THOC1’s functional role in GBM, we investigated its interaction with SIN3A, a histone deacetylase complex known to recruit HDAC1/2, key mediators of histone deacetylation [53]. Histone deacetylation plays a pivotal role in preventing excessive R-loop accumulation by rendering chromatin inaccessible to transcriptional machinery [54]. Therefore, enhanced HDAC activity provides a permissive chromatin environment in GBM cells, thereby amplifying the downstream impact of THOC1-mediated SIN3A recruitment. In other words, the elevated basal HDAC activity in GBM likely makes these cells more dependent on the THOC1–SIN3A axis for maintaining chromatin compaction and suppressing R-loop accumulation. We postulate that THOC1, through its interaction with SIN3A, prevents detrimental R-loop formation and enables GBM cell proliferation. Notably, prior studies have shown that elevated HDAC expression is implicated in glioblastoma progression and treatment resistance [55]. Furthermore, HDAC inhibition has demonstrated antitumor effects through decreased proliferation and increased DNA damage in both preclinical and clinical settings [56].

Epigenetic dysregulation, including heightened HDAC activity, is a hallmark of GBM [55,57] .In this context, THOC1-dependent recruitment of SIN3A–HDAC1/2 is predicted to exert stronger deacetylating and chromatin-compacting effects at target loci, thereby limiting R-loop accumulation and DNA damage. This dependency may help explain the greater genome-stability requirement—and potential therapeutic vulnerability—of the THOC1–SIN3A axis in GBM relative to normal cells.

We found that GBM cells display significantly greater levels of R-loops compared to non-cancerous cells, consistent with elevated transcriptional demand. We also observed robust THOC1-SIN3A interaction and greater histone deacetylation activity in GBM cells, suggesting that the elevated THOC1 may enhance SIN3A recruitment to maintain elevated R-loop homeostasis under oncogenic stress.

Our study identifies a functional link between THOC1 and SIN3A-mediated histone deacetylation, implicating the THOC1–SIN3A–HDAC1/2 scaffold as a key regulator of R-loop homeostasis in GBM. However, we recognize that the global loss of Class I HDAC activity observed using the pan–HDAC assay may appear broader than predicted by our proposed model. Our single-cell RNA-seq analyses from 63 GBM patients indicate that THOC1-high cells exhibit stronger SIN3A–HDAC1/2 scaffold activity, with weaker associations for other Class I HDAC complexes such as CoREST and NuRD, supporting relative specificity for the SIN3A–HDAC1/2 axis. Nonetheless, these transcriptomic associations do not substitute for direct biochemical validation. Future studies will address this limitation by employing complex-specific HDAC assays, chromatin occupancy mapping (CUT&RUN or CUT&Tag), and inducible SIN3A depletion to dissect whether THOC1 preferentially regulates HDAC1/2 enzymatic activity within the SIN3A complex or influences broader chromatin accessibility. These additional experiments will be essential to confirm the mechanistic specificity of THOC1-dependent HDAC regulation and to refine our understanding of how this pathway contributes to R-loop suppression and GBM progression [21].

Based on these findings, we examined THOC1’s influence on R-loop levels. We found that THOC1 knockdown increased R-loop levels across multiple GBM lines and reduced deacetylation activity. Conversely, overexpression in normal cells decreased R-loop levels and increased deacetylation activity, supporting the idea that THOC1 regulates R-loop formation by modulating the epigenetic landscape.

Building on these findings, we explored whether THOC1 selectively regulates specific networks of R-loop modulation. To explore this, we performed RNA-sequencing, which revealed that THOC1 selectively influences telomere packaging, suggesting a novel role in maintaining telomere stability. THOC1 knockdown led to increased telomeric R-loop levels, which, in turn, were also found to be associated with significantly reduced telomere length. Conversely, THOC1 overexpression in normal cells resulted in a decreased telomeric R-loop burden and significantly increased telomere lengths. These findings suggest that THOC1 not only controls cellular R-loops, but also telomeric R-loops, thereby playing a critical role in maintaining functional telomeres [3].

While our data show that the interaction of THOC1 and SIN3A regulates R-loops globally, the impact on glioblastoma may be disproportionately driven by its impact on telomeres. Telomeres are uniquely at risk for R-loop accumulation, as they are transcribed into telomeric repeat-containing RNA (TERRA), which base pairs with GC-rich regions of telomeres to form hybrids [38]. Subtelomeric regions remain transcriptionally active, whereas telomeric DNA contains heterochromatic histone modifications [58,59]. As a result, telomeres are predisposed to transcriptional conflict, which can result in R-loop accumulation and subsequent telomere instability and shortening [3]. We propose that THOC1 concentrates R-loop control at telomeres because these loci combine continuous GC-rich repeats transcribed into TERRA that readily base-pair with telomeric DNA, and a repressive chromatin environment enriched for H3K9me3/H4K20me3 and HP1, creating frequent transcriptional conflicts. This “perfect storm” makes telomeres disproportionately reliant on active hybrid suppression and histone deacetylation [58,60]. However, histone deacetylation restricts DNA accessibility and limits R-loop formation [45]. As a result, SIN3A-mediated HDAC recruitment by THOC1 could be especially important at telomeric regions where TERRA is transcribed and R-loop accumulation is predisposed. Our pathway analysis and experimental evidence showing that THOC1 knockdown increases telomeric R-loops and shortens telomeres provides a framework for understanding why telomeres are uniquely sensitive to THOC1 loss.

Mechanistically, we envision a two-arm model. THOC1 binds TERRA RNA to prevent and resolve telomeric R-loops [61], and recruits SIN3A–HDAC to maintain deacetylation and reduced polymerase access to prevent hybrid formation at telomeric DNA [62,63]. This framework is consistent with our data showing that THOC1 loss elevates telomeric R-loops and shortens telomeres, and with broader literature that telomeric R-loops drive fragility and instability unless controlled by RNase H–type activities and R-loop–suppressive factor [63].

Telomeric R-loops have drawn increasing attention for their role in genomic instability and tumor progression. Our data show that THOC1 depletion leads to telomeric R-loop accumulation, shortened telomeres, and GBM cell death. These findings align with studies linking dysregulated telomeric R-loops to telomere shortening and dysfunction across cancers, suggesting that THOC1 may be a promising target to disrupt telomere stability in GBM. Additionally, our data also show a potential mechanism of THOC1 overexpression in the transformation into a cancerous phenotype. Perhaps this is achieved through decreased telomeric R-loops, which studies have previously shown to be involved in oncogenic transformation by inhibiting telomerase. However, this mechanism needs further exploration.

Glioblastoma remains one of the deadliest cancers, with poor outcomes despite therapeutic advances. Through a comprehensive CRISPR-Cas9 knockout screen, we identified approximately 150 previously unrecognized genes crucial for GBM progression. THOC1 was found to be significantly elevated in GBM and particularly responsible for promoting GBM aggressiveness. Our study sheds light on the intricate landscape of GBM progression and oncogenesis by revealing the critical role of THOC1. Previously, the precise workings of R-loops in GBM remained elusive; however, our findings illuminate how THOC1 maintains the delicate R-loop equilibrium to sustain replicative potential without leading to DNA damage. By uncovering the epigenetic mechanisms through which THOC1 controls R-loop homeostasis, particularly including telomere maintenance, this study presents new insight into GBM biology and therapeutic vulnerability. Therapeutically targeting THOC1-SIN3A axis, either through chemical optimization of the luteolin scaffold or design of small-molecule and peptide disruptors, will be essential for successful clinical translation of this approach. Such approaches may selectively destabilize the delicate R-loop landscape and promote telomere dysfunction, thereby inducing GBM cell death and interrupting a key axis of GBM progression.

## Ethics approval and consent to participate

All intracranial PDX experiments were approved by the IACUC (IS00004080 and IS00021383) and conducted in an AAALAC-accredited facility in accordance with the NIH Guide. For intracranial models, the maximal permitted tumour burden is defined by IACUC-approved humane endpoints. Mice were euthanized at the first occurrence of any endpoint, including ≥20% body-weight loss from baseline, body-condition score ≤2/5, unrelieved or recurrent seizures, severe neurological deficit, inability to eat/drink, or moribund state. These limits were not exceeded in any experiment.

## Data availability

All data supporting the findings are available within the article and/or its Supplementary Materials. Public datasets used include TCGA (https://www.cancer.gov/tcga), Human Protein Atlas, and GBMSeq. Single-cell sequencing data generated in this study have been deposited in GEO under accession GSE227348.

## Consent for publication

Not applicable.

## Code availability

Custom code used for the bioinformatic analyses is available from the corresponding author upon reasonable request.

## Financial support

1R01NS096376, 1R01NS112856 and P50CA221747 SPORE for Translational Approaches to Brain Cancer (to A.U.A.); 5R01NS110703, R01CA245969 and U19CA264338 (to A. M. S.); DP2AI158157 and R21AI144417 (to A. B).

## CRediT authorship contribution statement

**Shreya Budhiraja:** Conceptualization, Data curation, Investigation, Methodology, Visualization, Writing – original draft. **Umme H. Faisal:** Data curation, Writing – original draft. **Shivani Baisiwala:** Conceptualization, Investigation, Methodology, Visualization. **Rafal Chojak:** Data curation, Software, Validation, Writing – original draft. **Lara Koutah:** Data curation, Writing – original draft. **Noah B. Drewes:** Data curation, Writing – original draft. **Sia Cho:** Data curation. **Hasaan A Kazi:** Data curation. **Rebecca Chen:** Data curation, Writing – original draft. **Ella N Perrault:** Data curation, Writing – original draft. **Li Chen:** Data curation, Writing – original draft. **Cheol H. Park:** Data curation, Writing – original draft. **Maeve C. O’Shea:** Data curation, Writing – original draft. **Khizar Nandoliya:** Data curation, Writing – original draft. **Joseph T. Duffy:** Data curation, Software, Validation, Writing – original draft. **Peiyu Lin:** Data curation. **Adam M Sonabend:** Writing – review & editing. **Crismita C. Dmello:** Writing – review & editing. **Atique U. Ahmed:** Conceptualization, Methodology, Supervision, Writing – review & editing.

## Declaration of competing interest

The authors declare that they have no known competing financial interests or personal relationships that could have appeared to influence the work reported in this paper.

## Appendix Supplementary materials

Image, application 1
