# Supplementary material for: THOC1 complexes with SIN3A to regulate R-loops and promote glioblastoma progression
Source: Neoplasia. 2026 Jan 6;72:101271. doi: 10.1016/j.neo.2025.101271 (PMC12808535; doi:10.1016/j.neo.2025.101271)
Supplement: Supplementary file 1 [file mmc1.docx]

**THOC1 complexes with SIN3A to regulate R-loops and promote glioblastoma progression**

**Supplementary Material**

**METHODS AND MATERIALS**

*CRISPR-Cas9 knockout screen*

The screening procedure closely followed previously established protocols [1]. We initiated the process by infecting H4 human GBM cells with the Brunello whole-genome knockout library (Addgene, Cambridge, MA, USA), which included around 19,000 genes, each with 4 sgRNAs, along with 10,000 sgRNA non-targeting controls. To prepare the library, we cultured 80% confluent HEK293T cells in a T225 flask for 20-24 hours. Subsequently, we introduced Opti-MEM I reduced serum, psPAX (10.4 µg/ml), pMD2.G (5.2 µg/ml), and Lipofectamine Plus reagent to the cells. After a 4-hour incubation, we filtered the media using 0.45 µM filters and stored the virus at -80 °C.

To determine the virus titer, we seeded 3 million H4 cells and 2 ml of media in a 12-well plate. We then added varying volumes of virus (ranging from 25 ul to 400 ul) along with 8 µg/µl of polybrene to each well, followed by spinfection at 1000 g for 2 hours at 33 °C. After a 24-hour incubation at 37 °C, we harvested the cells, seeded them at 4000 cells per well, and cultured them for 96 hours alongside non-transduced cells. Finally, we conducted a titer glo assay to assess cell viability and determine the multiplicity of infection (MOI).

Next, we utilized 70,000 sgRNAs to culture and spinfect 500 million H4 cells. Following a 4-day selection period with 0.6 µg/ml puromycin, 150 million cells remained viable. We extracted genomic DNA (gDNA) from 50 million of these cells after amplifying sgRNA with unique barcoded primers serving as controls. The remaining cells were expanded to 200 million over a 4-day period before undergoing treatment with 700 uM DMSO and TMZ for 14 days. Subsequently, we harvested the cells to amplify sgRNA and create the sequencing library.

The gDNA extraction was carried out using the Zymo Research Quick-DNA Midiprep Plus Kit (Cat no: D4075, Irvine, CA, USA), followed by purification with 100% ethanol containing 1/10 volume of 3 M sodium acetate (PH 5.2), and the addition of a 1:40 glycogen co-precipitant (Invitrogen Cat no: AM9515). To measure the gDNA concentration, we employed NanoDrop 2000 (Thermo Scientific, Waltham, MA, USA) and performed PCR for DNA amplification.

For sequencing, we used a NextSeq machine, generating 300 million reads for the four sgRNA pools, each with 1000 reads/sgRNA. The sequencing process consisted of 80 cycles of read 1 (forward) and 8 cycles of index 1, as per the Illumina protocol. To enhance coverage, we introduced 20% PhiX into the NextSeq.

To analyze the computational data, we utilized CRISPRAnalyzR and the CaRpools pipeline. The significance of the changes was determined using DESeq2 and the MaGeCK algorithms.

*Cell lines and culture*

Patient-derived xenograft (PDX) glioma cell lines (GBM43, GBM6) were obtained from C.D. James at Northwestern University and maintained according to the published protocol [2]. In this case, the PDX cells were cultured in Dulbecco’s Modified Eagle’s Medium (DMEM) supplemented with 1% fetal bovine serum (FBS; Atlanta Biologicals, Lawrenceville, GA, USA) and 1% penicillin-streptomycin (Cellgro, Herdon, VA, USA; Mediatech, Herndon, VA, USA).

Human glioma cell lines, U251 and H4, were procured from the American Type Culture Collection (ATCC; Manassas, VA, USA). These cells were cultured in DMEM containing 10% FBS and 1% penicillin-streptomycin mixture. A frozen stock was used to replenish cells that had been expanded for a maximum of 4 passages. These frozen stock cells were stored at -180C in liquid nitrogen in pure FBS containing 10% DMSO (dimethyl sulfoxide).

Neural stem cell, astrocyte, and fibroblast lines were acquired from American Type Culture Collection (Manassas, VA, USA) and from Kerafast (Boston, MA, USA). H1B.F3 and fibroblast (WI-38) lines were cultured in DMEM supplemented with 10% FBS and 1% penicillin-streptomycin. NSC LM008 cells were cultured in neurobasal media (ThermoFisher Scientific; San Jose, CA, USA) and supplemented with B27 (no Vitamin A; Invitrogen, Carlsbad, CA, USA), N2 (Invitrogen), 1% penicillin-streptomycin, basic fibroblast growth factor (bFGF, 10ng/mL; Invitrogen), and epidermal growth factor (EGF, 10ng/mL; Invitrogen). To ensure optimal growth, the media was replaced every 2 days.

*Animals and in vivo models*

Athymic nude mice (nu/nu; Charles River, Skokie, IL, USA) were used in this study and housed in accordance with Institutional Animal Care and Use Committee (IACUC) guidelines. All applicable federal and state statutes governing the use of animals for biomedical research were also in compliance with this study.

Intracranial implantation of GBM cells was performed as previously described in our laboratory’s established GBM mouse model [3]. Animals initially received a prophylactic intraperitoneal (i.p.) injection of Buprenex and Metacam, followed by an anesthetizing i.p. injection of a ketamine/xylazine mixture (Henry Schein; New York, NY, USA). Sedation was confirmed by a toe-pinch test. Artificial tears were applied to their eyes, and the scalp was sterilized using Betadine and ethanol. Using a scalpel, the skull was exposed, at which point an approximately 1-mm burr hole was drilled above the right frontal lobe. The mice were then placed into a stereotactic rig where they received an injection of 150,000 GBM PDX cells using a Hamilton syringe 3mm from the dura, for a duration of 1 minute. To ensure proper release of the cell suspension, the needle was slightly raised and left undisturbed for 1 additional minute. Careful removal of the needle followed, and at this point the animal’s head remained in position while the skin of the scalp was sutured closed (Ethicon; Cincinnati, OH, USA).

Drug treatment regimens were initiated 7 days after intracranial implantation. I.p. injections of either TMZ (2.5mg/kg) or equimolar DMSO were delivered to each animal daily for 5 consecutive days. Following IACUC and Northwestern University guidelines, animals were sacrificed once it was evident that they would not survive past the following morning.

*Cellular transfection*

To generate lentiviral particles, low-passage X293 cells (ATCC; Manassas, VA, USA) were plated at 80% confluency based on a previously described protocol [2]. Six hours after plating, the cells were transfected with a mixture of HP DNA Transfection Reagent (Sigma-Aldrich; St. Louis, MO, USA) diluted in Opti-MEM medium (Gibco; Waltham, MA, USA), along with packaging and target plasmids, in accordance with the manufacturer’s instructions (Addgene).

*Viral transduction*

48-72 hours after maintaining the cells following transfection, the virus supernatant was harvested and sterilized using a 45-µm filter, and then ultracentrifuged at 133,897 relative centrifugal force (RCF) for 2 hours to isolate viral particles. The resulting viral pellet was resuspended in phosphate-buffered saline (PBS) and aliquoted for storage in -80 °C until use. When ready for cellular transduction, cells were resuspended in 50 µL of media, and 20 MOI lentivirus amounts were added per sample, along with 1 mL of appropriate media. This virus-media mixture was spun for 2 hours at 37 °C at 850g. Following centrifugation, the cells were plated with fresh media along with the viral media in which they were spun. 48-72 hours later, the media was changed to non-viral containing media, and the efficiency of the transduction was assayed via Western blot.

*Cell viability assays*

Viability assays were performed according to a previously established protocol [4]. Cells were plated at 5000 per well in a 96-well plate with eight replicates per condition. Cells were allowed to grow and attach for 24 hours before treatments began. At this time, TMZ was administered using our laboratory’s standard dose-response protocol [2]. Following the assigned hours of treatment, the medium was removed and 110µL of MTT solution was instead added to the cells and the plate was then incubated at 37C for 5 hours. The MTT solution was prepared by diluting MTT stock reagent at 5 mg/mL in PBS. This mixture was diluted in fresh media at a 1:10 ratio before being added to each well. Post-incubation, the medium was carefully removed, and the cells were resuspended in 100 µL of DMSO to dissolve any crystals that had formed. After 10 minutes at room temperature, the place was loaded into a plate reader at an absorbance of 570nm and the data was analyzed to find percent viability in each well.

*Immunofluorescence*

Mouse brains were harvest and frozen in cryoprotectant on dry ice, stored at -80C in 8µm sections, and stained according to standard immunohistochemistry protocol [2]. Sections were thawed at room temperature for 15-20 minutes and washed two times for 5 minutes in PBS + 0.05% Tween 20 (PBS-T) to remove residual cryoprotectant. The immunopen was then used to circle each brain section. Each section was fixed in ~100 μL of 4% PFA (ThermoFisher Scientific; Rockford, IL, USA) at room temperature for 15 minutes and washed twice, each time for 5 minutes. Following this, antigen retrieval was performed by first boiling the samples in sodium citrate buffer for 20 minutes, then a 30-minute cooling period at room temperature. Slides were then washed 3 times for 5 minutes in PBS-T. Blocking was achieved by incubating slides in a 10% bovine serum albumin (BSA) solution with Triton-X (ThermoFisher Scientific; Rockford, IL, USA) for 2 hours at room temperature. Next, slides were incubated at 4 °C overnight with 100µL primary antibody solutions diluted in 1% BSA Triton-X. In the morning, the slides were washed 3 times for 10 minutes in PBS-T. 100 µL of secondary antibodies diluted in 1% BSA and Triton-X were then applied and incubated for 2.5 hours at room temperature. Lastly, they were washed in PBS-T 3 times for 10 minutes. To image these slides, a drop of ProLong Gold Antifade reagent with 4’,6-di-amidino-2-phenylindole (DAPI) was added to each section (ThermoFisher Scientific; Rockford, IL, USA). Images of these slides were organized and assessed in ImageJ.

Additional experiments were conducted following the immunocytochemistry protocol previously published [5]. After removing the chamber slides from incubation and washing them once with PBS, 200 µL of 4% PFA was added to each well and allowed to incubate at room temperature for 10 minutes. The slides were then washed gently with PBS. Then, the cells were blocked for 2 hours at room temperature with 200 µL of a 10% BSA solution. After aspirating the BSA off the slides, 100 µL of primary antibody diluted in 1% BSA and Triton-X was added, and the chamber was then incubated overnight at 4 °C. The next morning, the wells were washed 3 times for 5 minutes using 1% BSA + Triton-X. After this, 200 µL of secondary antibody in 1% BSA and Triton-X was added and incubated at room temperature for 2 hours. Following incubation, the wells were washed 3 times for 10 minutes with PBS. Finally, ProLong Gold Antifade reagent with DAPI was added to each section to allow for imaging using a Leica microscope. These images were analyzed in ImageJ.

*Flow cytometry*

Cells containing the plasmid of interest were harvested and spun down to a pellet, then washed with 100µL of PBS. Primary antibodies in FACS buffer (50µL per well) were added at room temperature in the dark and incubated for 1 hour. Cells were then spun down and washed once more with 100µL of PBS. Following this, the cells were resuspended in 80µL of FACS analysis buffer and spun down again. 100µL of fix-perm buffer was added to each well. This buffer was prepared in a 1:3 ratio of fix/perm to buffer. Samples were then incubated at room temperature in the dark for 20 minutes. At this time, a 1:10 ratio of fix perm buffer was prepared in double-distilled water. 100µL of this solution was subsequently added on top of fix/perm. A 10-minute incubation period at room temperature followed. Next, the samples were spun down for 5 minutes at 1500 rpm and washed with a 1:10 perm buffer solution. The primary antibody was added to the perm buffer solution and 50µL was added to each well and allowed to incubate overnight at 4C. The next day, the samples were washed 3 times with FACS buffer and a secondary antibody contained in FACS buffer was added to them. The samples were incubated for 1 hour at room temperature. Finally, the samples were washed and resuspended in 100µL of FACS buffer and analyzed on the BD LSRFortessa Cell Analyzer.

*BrDu Flow Cytometry Assay*

Cells were treated with DMEM that contained 10 um/ml concentration of 5-Bromo-2’-deoxyuridine (BrDu). Cells were pulsed and then placed in the incubator overnight. 24 hours later, cells were washed three times with 1x DPBS and sat in the DPBS for 5 minutes for each wash. Cells were then trypsinized and collected in 15 ml centrifuge tubes, before being spun at 1200 RPM for 5 minutes at 4 degrees Celsius to pellet the cells. Cells were washed an additional two times with PBS before being transferred to Fisherbrand 1.2 mL Micro Titer Tubes (catalog no. 02-681-386) in 300 ul DPBS. While the tubes were being vortexed, 700 ul of ice cold 70% ethanol was added to the tubes. Tubes then sat at room temperature for 30 minutes to fix the cells. After the 30 minute incubation, cells were washed twice with cold DPBS. 100 ul of DNAse buffer was then added to each tube, and the cell pellet was resuspended before being left to sit for an hour at room temperature. After the incubation cells were suspended in FACS buffer (40 ml 1x DPBS, 800 ul FBS) to neutralize the DNAse, and then washed one more time in this buffer. Cells were then stained with an unconjugated primary BrDu antibody (Cell signalling catalog no. BrdU5292S) at a 1:200 antibody to Flow Cytometry Staining Buffer (Invitrogen Thermo Fisher ebiosience catalog no. 00-4222-57) dilution ratio. Cells were left to incubate for an hour. Cells were then washed twice with FACS buffer. Cells were then stained with a FITC conjugated secondary antibody (Bioss catalog no. bs-0296G-FITC) at a 1:400 ratio in Flow Cytometry antibody buffer. Cells were left to incubate for 30 minutes. Cells were washed twice with FACS buffer being analyzed on the A5 FACSymphony A5.2 Spectral Analyzer.

*Western blotting*

Cells were trypsinized, washed with PBS, and resuspended in mammalian protein extraction reagent (M-PER; ThermoFisher Scientific; Rockford, IL, USA) according to the protocol [6]. M-PER was supplemented with protease and phosphatase inhibitor cocktail (PPI’ ThermoFisher Scientific; Rockford, IL, USA) and EDTA (ThermoFisher Scientific; Rockford, IL, USA). Cells were vortexed for 1 minute 3 times with 10 minutes of rest on ice between each vortex. The resulting lysate solutions were centrifuged at 13,000rpm for 10 minutes at 4C. The supernatant was carefully collected, and the resultant protein concentration was determined via bicinchoninic acid assay (ThermoFisher Scientific; Rockford, IL, USA). This concentration was used to specify the amount of lysate required to make each Western blot sample. Each sample comprised of equal amounts of protein and varying amounts of SDS buffer (SDS sample buffer; Alfa Aesar; Ward Hill, MA, USA) with M-PER, resulting in equal total volumes across the samples. After a brief period of vortexing, the samples were boiled at 95 °C for 10 minutes and then stored at -20 °C until further use.

Samples were run through 8% SDS-polyacrylamide (SDS-PAGE, made in-house) via by gel electrophoresis (Bio-Rad; Hercules, CA, USA). The proteins were then transferred onto 0.45-μm polyvinylidene difluoride membranes (Milipore; Darmstadt, Germany). After transferring, the membranes were washed 3 times for 10 minutes in PBS, then blocked for 2 hours with tris-buffered saline (TBS), comprised of 5% powdered nonfat milk and 0.05% Tween 20 (Sigma-Aldrich; St. Louis, MO, USA). Next, the membranes were washed again three times for 10 minutes in TBS-T and subsequently placed in primary antibody solutions composed of a 5% BSA solution with sodium azide. The blots were then incubated overnight on a shaker at 4 °C. The next morning, the membranes were washed three times in TBS-T for 10 minutes each and then placed in secondary antibody diluted 1:4000 in 5% milk. After an additional round of TBS-T washes, the membranes were coated in enhanced chemiluminescence (ECL, Clarity ECL, Bio-Rad). These membranes were developed using the ChemiDoc Imaging System (Bio-Rad).

*Dot blotting*

To detect R-loops, cultured cells were trypsinized, pelleted, and washed with PBS to remove residual culture medium, followed by a previously validated protocol [7]. Briefly, the harvested cell suspension was transferred to 1.5 mL tubes. Subsequently, cold cell lysis buffer was added to the cell pellet at a rate of 300 μL per 2 × 10^6 cells, and thorough resuspension was achieved through pipetting. The cells were incubated on ice for 10 minutes and then centrifuged at 500 × g for 5 minutes to pellet the nuclei. The supernatant was discarded, and the nuclear pellet was resuspended in 400 μL of cold nuclear lysis buffer, followed by an additional 10-minute incubation on ice.

The subsequent purification of genomic DNA, which included RNA-DNA hybrids, commenced with the addition of 3 μL of 20 mg/mL proteinase K and an incubation period of 3–5 hours at 55 °C. The purification steps included DNA extraction using phenol:chloroform:isoamyl alcohol, precipitation, ethanol washing, and air drying. The resultant pellet was resuspended in 12 μL of elution buffer and further incubated for 30 minutes at 37 °C. The DNA concentration was subsequently measured using spectrophotometry. Nucleic acid samples were then diluted to desired concentrations in elution buffer. Positively charged nylon membranes were prepared, allowing 2 μL of each sample to be spotted. The samples were allowed to saturate into the membrane for at least 2 minutes before proceeding to UV crosslinking.

The membranes were incubated in blocking solution (5% milk in Tris-buffered saline with 0.05% Tween-20) for 1 hour at room temperature to minimize nonspecific binding. Subsequently, the membranes were incubated overnight in primary antibodies (in 5% milk in TBST) at 4 °C with shaking. Specifically, anti-dsDNA antibody (1:10,000 dilution) was added to one membrane, while the other received 1 μg/mL S9.6 antibody (1:1,000 dilution). Following incubation, the membranes underwent primary antibody removal and were washed three times with TBST for 5–10 minutes each. The membranes were then incubated with horseradish peroxidase (HRP)-conjugated secondary antibody (anti-mouse, 1:5,000 dilution) in 5% milk in TBST with shaking at room temperature. After secondary antibody incubation, the membranes underwent three additional washes with TBST for 5–10 minutes each. Signal detection was achieved through ECL reagents, followed by signal intensity quantification using standard image processing tools such as ImageJ [7].

*Immunoprecipitation*

Protein samples were isolated and normalized following the details above. A mixture of 5uL of ubiquitin antibody was incubated with 100µg of protein and protease/phosphatase inhibitors in Eppendorf tubes and placed in a rotary shaker in a cold room overnight. The next day, 30µL of Protein A/G beads were added to the sample and incubated for 2 hours at room temperature. Reactions were washed 3 times with 500µL of M-PER and centrifuged at 3200g for 5 minutes. After the third wash, 50µL of supernatant was reserved and 50µL of 2X SDS was added to all reactions. Samples were then incubated at 55C for 10 minutes to elute and were subsequently spun down again at 3200g for 5 minutes to separate the beads from the supernatant. Once the supernatant was collected, it was boiled for 10 minutes at 95C and loaded directly onto an SDS-PAGE gel. The remaining protocol was followed according to the Western blot protocol above.

*HDAC Activity Assay*

The HDAC activity assay utilized an Abcam kit (ab156064, Cambridge, UK). Briefly, cells were gathered, and the lysate was obtained according to the manufacturer's instructions. A black 96-well plate was prepared with the lysate and the provided kit buffers. Once the reaction commenced, the plate underwent continuous readings every 1-2 minutes using a plate reader.

*Telomere Isolation*

Telomere isolation was utilized the TeloTAGGG™ kit (#12209136001, Millipore Sigma). Briefly, cells were gathered, and the genomic isolate was obtained according to the manufacturer's instructions. Digestion of genomic DNA was then conducted using provided restriction enzymes, resulting in digestion of non-telomeric DNA and preservation of telomeric DNA and sub-telomeric DNA. Telomeric isolates were then utilized for further experiments.

*Quantitative PCR*

Nucleic acid extraction was initially carried out according to previously discussed protocols. Following a 1:10 dilution of the isolated DNA in distilled water, reactions were arranged in triplicates, incorporating standardized quantities of DNA, SyberGreen (Thermo Fisher, Rockford, IL, USA), as well as forward and reverse primers (IDT, Newark, NJ, USA), which were intended for subsequent quantitative PCR analyses. The outcomes were recorded using a conventional qPCR apparatus. All primer sequences were generated using Primer-BLAST.

*Single-cell RNA Sequencing Analysis*

Single-cell RNA sequencing data were analyzed using Seurat (v4) in R. We loaded the integrated Seurat object and filtered it to include only GBM cells treated with DMSO (control). Cells co-expressing THOC1 and SIN3A were identified based on specific expression thresholds and labeled accordingly. The data were normalized and scaled to regress out cell cycle phases, total RNA counts, and feature counts. Next, we selected 2,000 variable features using the "vst" method and performed dimensionality reduction with PCA and UMAP based on the top 10 principal components. Differential expression analysis between co-expressing and non-coexpressing cells identified marker genes, which were visualized using violin plots and heatmaps. Gene set enrichment analysis (GSEA) was conducted with MSigDB C2 gene sets via the clusterProfiler package and illustrated with paired dot plots. We calculated signature scores using the UCell package and visualized them across groups with dot plots. The Seurat object (GBMap) was imported. For targeted gene expression analysis, genes of interest (THOC1 and SIN3A) were identified and grouped by multiple Ensembl IDs per gene. The aggregated gene expressions were integrated into the RNA assay, followed by normalization using the LogNormalize method and scaling to a scale factor of 10,000. Cells were categorized based on the expression levels of THOC1 and SIN3A (> 0). These categories were visualized using UMAP plots with distinct color codings to highlight co-expression patterns. All statistical analyses and visualizations were performed in RStudio.

Analyses used GBmap (IDH-wildtype GBM atlas) restricted to malignant cells. Drivers were THOC1 (primary), SIN3A, and SIN3B. The primary readout was a CORUM-anchored SIN3A–HDAC1/2 complex core (gene list below). Controls were CoREST and NuRD cores and NON-OVERLAP variants (constructed by removing any genes shared with the SIN3A core). We also scored MSigDB HDAC inhibitor response sets (MARKS_HDAC_TARGETS_UP and MARKS_HDAC_TARGETS_DN) retrieved via msigdbr (human collections). Module scores were computed as the mean normalized expression of member genes. For each patient, cells were ranked by driver expression and binned into quartiles (Q1–Q4) or tails (LOW/HIGH at 5/10/20%). Within each patient we computed Δ as Q4–Q1 (quartiles) or HIGH–LOW (tails). Patients contributed if both bins met the minimum-cells criterion (canonical: 10% tails, ≥20 cells/bin). Patient-level Δ values were meta-analyzed using random-effects (DerSimonian–Laird τ²) with Hartung–Knapp confidence intervals. Independently, we estimated patient-level partial correlations between driver expression and module score after regressing out library size and G1/S & G2/M proliferation; Fisher-z values were meta-analyzed with HK CIs. To test whether effects exceeded what could be achieved by arbitrary gene sets of similar abundance, we compared the driver→module association against a null distribution built from size/abundance-matched random gene sets (two-sided empirical p). We also benchmarked against CoREST/NuRD and their NON-OVERLAP variants to probe scaffold-level specificity. For each driver, p-values across modules (Δ-meta, partial-r meta, specificity) were BH-adjusted.

Module definitions: Primary readout - SIN3A complex core (CORUM-anchored; <https://pubmed.ncbi.nlm.nih.gov/30357367/> ): SIN3A, HDAC1, HDAC2, RBBP4, RBBP7, SAP30, SAP30L, SAP130, SUDS3, ARID4A, ARID4B, ING1, ING2, BRMS1, BRMS1L. Control complexes: CoREST core: RCOR1, RCOR2, RCOR3, KDM1A, HDAC1, HDAC2, PHF21A, HMG20B NuRD core: CHD4, MTA1, MTA2, MTA3, HDAC1, HDAC2, RBBP4, RBBP7, MBD2, MBD3, GATAD2A, GATAD2B Specificity (NON-OVERLAP variants; remove any genes shared with SIN3A core) COREST_CORE_NOOVLP: RCOR1, RCOR2, RCOR3, KDM1A, PHF21A, HMG20B NURD_CORE_NOOVLP: CHD4, MTA1, MTA2, MTA3, MBD2, MBD3, GATAD2A, GATAD2B.

*Statistical analysis*

Statistical analyses were performed and represented using GraphPad Prism v9.0 software (GraphPad Software; San Diego, CA, USA). Generally, data are presented as mean with standard deviation for continuous variables and number or percentage for categorical variables. Differences between the two groups were assessed using Student’s *t* test or the Wilcoxon rank sum test, where applicable. Differences between multiple groups were assessed using ANOVA with *post* *hoc* Tukey’s test, or Mann-Whitney *U* test, followed by Bonferroni correction as appropriate. Survival curves were generated using the Kaplan-Meier method and compared using the log-rank test. All tests were two-sided, and p<0.05 was considered statistically significant. *In vitro* experiments were performed in biological triplicate.

**SUPPLEMENTARY FIGURE**

**Supplementary Figure 1**


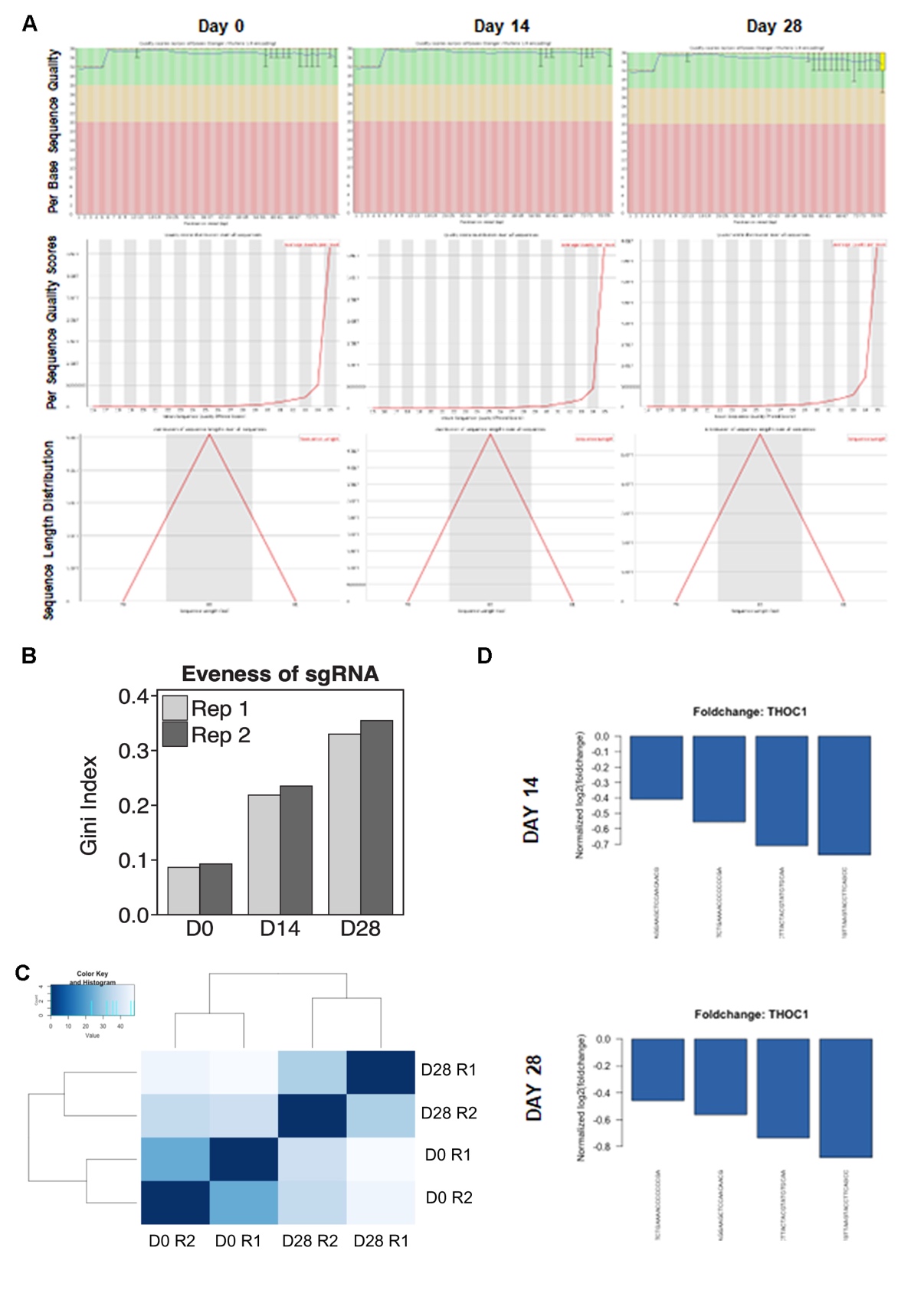


**Figure S1:** *CRISPR-Cas9 screen quality* ***A)*** Quality assessment of sequencing data, displaying per base sequence quality, sequence quality scores, and length distribution to validate screen results. ***B)*** Distribution of gene read counts and sgRNA frequencies across conditions measured by the Gini Index. ***C****)* Principal component analysis (PCA) reveals the consistency of replicates and differences in condition, supporting the overall quality of the screen. ***D****)* Performance of the individual sgRNA against THOC1 at days 14 and 28 of our screen.

**Supplementary Figure 2**


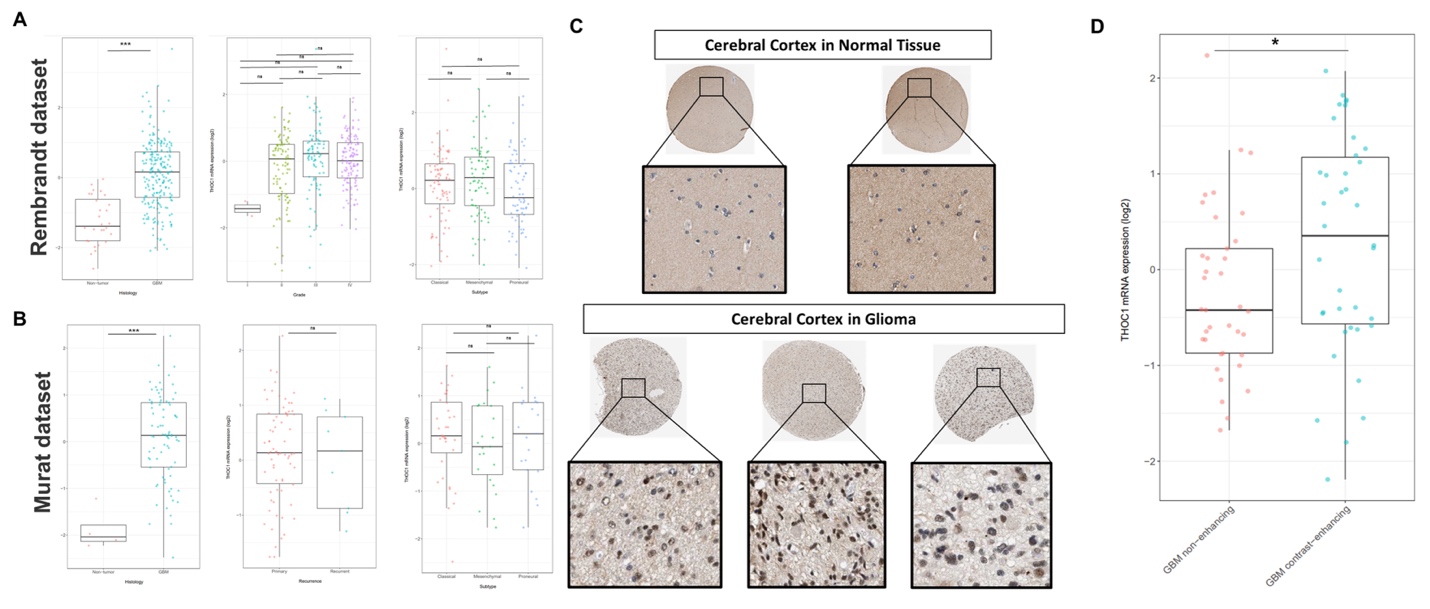


**Figure S2:** *THOC1 expression in patient samples* ***A***) Comparative analysis of THOC1 RNA expression across tumor grades, recurrence status, and subtypes showed no significant correlation, based on data from the GlioVis portal in the Rembrandt dataset. ***B)*** A comparative analysis of THOC1 RNA expression across tumor grades, recurrence status, and subtypes revealed no significant correlation, based on data from the GlioVis portal in the Murat dataset. ***C)*** Qualitative assessment of THOC1 protein expression in GBM tissue compared to normal brain tissue from the Protein Atlas. ***D)*** THOC1 RNA expression in non-enhancing versus contrast-enhancing regions of GBM from the GlioVis portal.

**Supplementary Figure 3**
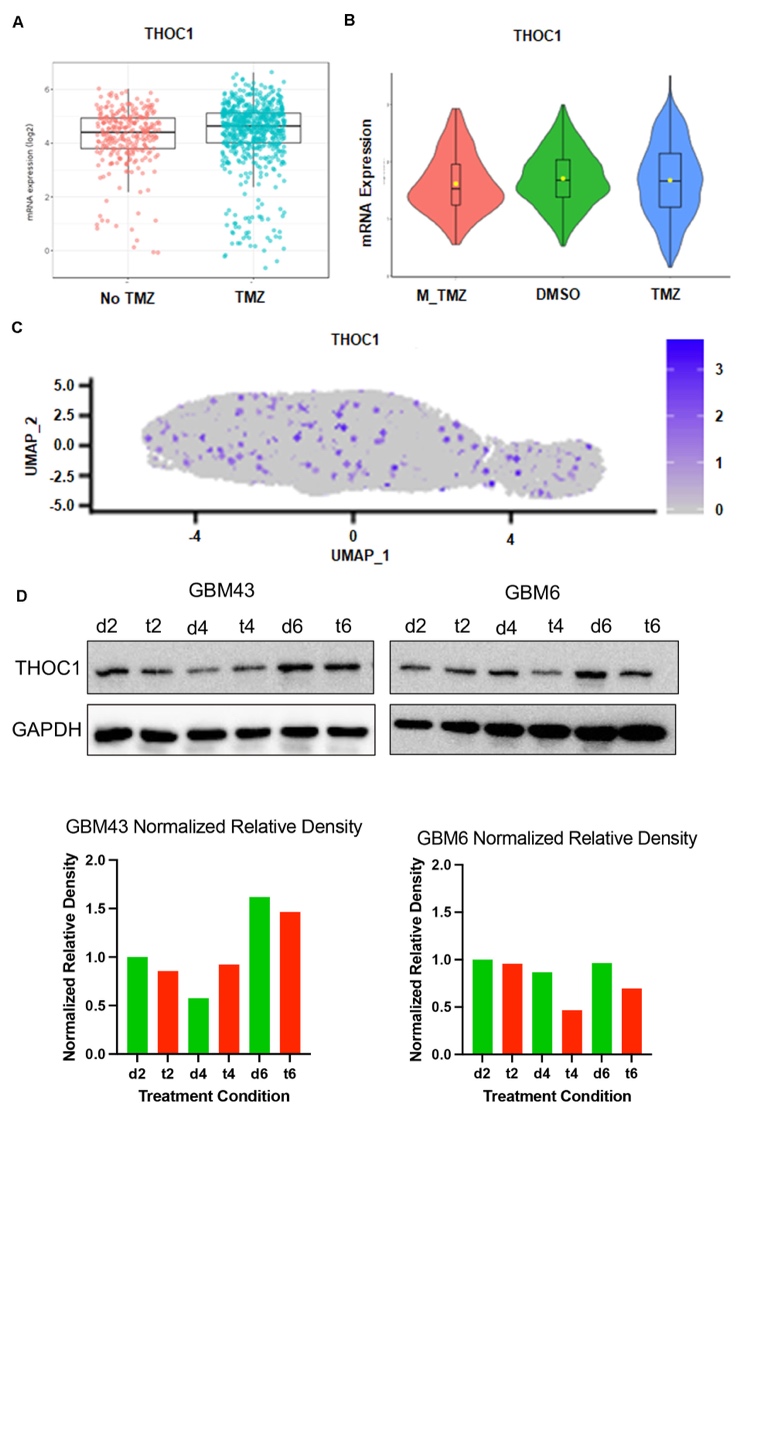


**Figure S3:** *THOC1 Expression During TMZ Therapy in GBM* ***A)*** GlioVis portal analysis showing no increase in THOC1 RNA expression in GBM patients treated with temozolomide (TMZ) compared to those not treated. ***B)*** Violin plot of single-cell RNA sequencing data comparing THOC1 expression across mid-therapy TMZ (M_TMZ), DMSO, and post-TMZ conditions, demonstrating no significant differences in THOC1 levels across treatment groups ***C)*** UMAP projection of single-cell RNA sequencing data showing uniform THOC1 expression across treatment conditions. D) Western blot analysis of GBM43 and GBM6 cells at multiple time points (days 2–6) following DMSO (D, green) or TMZ (T, red, 50 μM) treatment, showing consistent THOC1 expression over time.

**Supplementary Figure 4 said figure 3 before changed it to figure 4**


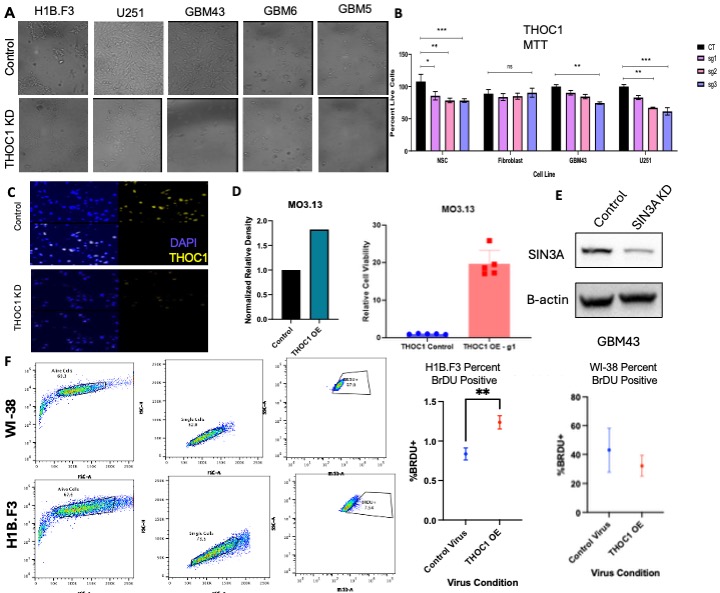


Figure S4: THOC1 Knockdown Reduces Viability and Proliferation in GBM Cells A) Representative images showing decreased cell density in THOC1 KD H1B.F3, U251, GBM43, GBM6, and GBM5 cells compared with controls. B) Verification of decreased cell viability in THOC1 knockdown GBM cell lines through MTT assays showing reduced proliferation in THOC1-knockdown U251 and GBM43 cell lines, compared to knockdown fibroblasts. C) Immunohistochemical staining confirming THOC1 knockdown in brain tissue from mice implanted with THOC1-knockdown GBM43 cells. D) Western blot densitometry and cell viability analysis of MO3.13 oligodendrocyte progenitor cells overexpressing THOC1, demonstrating enhanced survival. E) Western blot confirming SIN3A knockdown in GBM cells. F) BrdU incorporation flow cytometry confirming a significant increase in S-phase (BrdU⁺) cells in H1B.F3 following THOC1 overexpression, consistent with enhanced proliferative activity.

**Supplementary Figure 5**


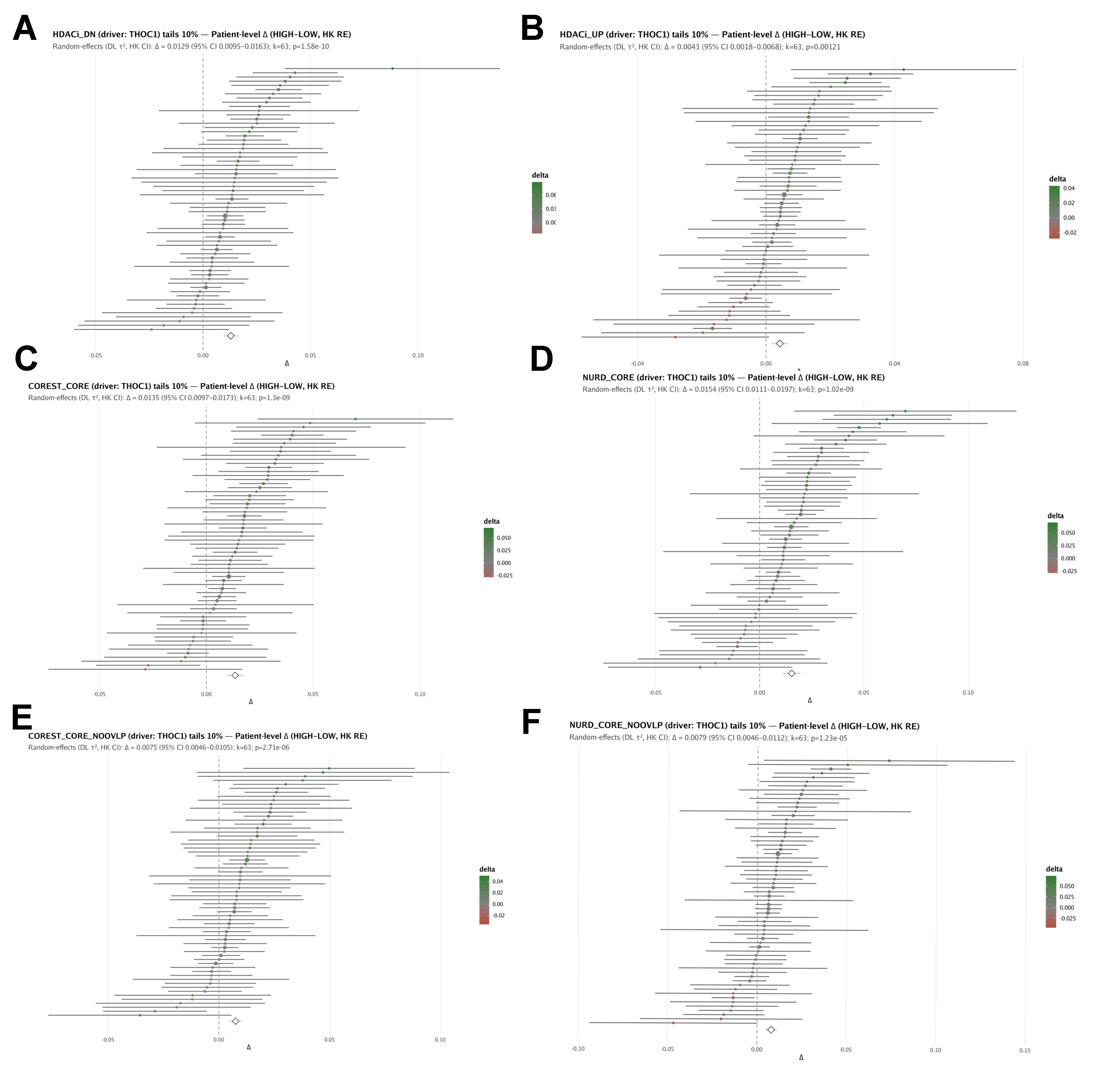


Figure S5: Scaffold specificity across HDAC1/2 complexes and modulation of HDAC-inhibitor response signatures. A–B) THOC1 effects on CoREST and NuRD cores (10% tails; ≥20 cells/bin) are significant (HK 95% CIs shown). C–D) NON-OVERLAP variants-constructed by removing any genes shared with the SIN3A-core-show attenuated effects: CoREST Δ 0.0135 → 0.0075, NuRD Δ 0.0154 → 0.0079 (all with k = 63; exact CIs and p in panel subtitles). Persistence with attenuation in NON-OVERLAP variants argues against a purely generic HDAC1/2 signal and supports SIN3A-scaffold–centered association. E) Random-effects forest for THOC1 → HDACi_DN (MSigDB MARKS_HDAC_TARGETS_DN) Δ = 0.0129, k = 63, p = 1.58×10⁻¹⁰. F) Random-effects forest for THOC1 → HDACi_UP (MSigDB MARKS_HDAC_TARGETS_UP): Δ = 0.00430, k = 63, p = 1.21×10⁻³ . Both HDAC-inhibitor response signatures are significantly modulated in the canonical within-patient contrast. Panel subtitles report Δ, 95% CI, k, and p.

**Supplementary Figure 6**


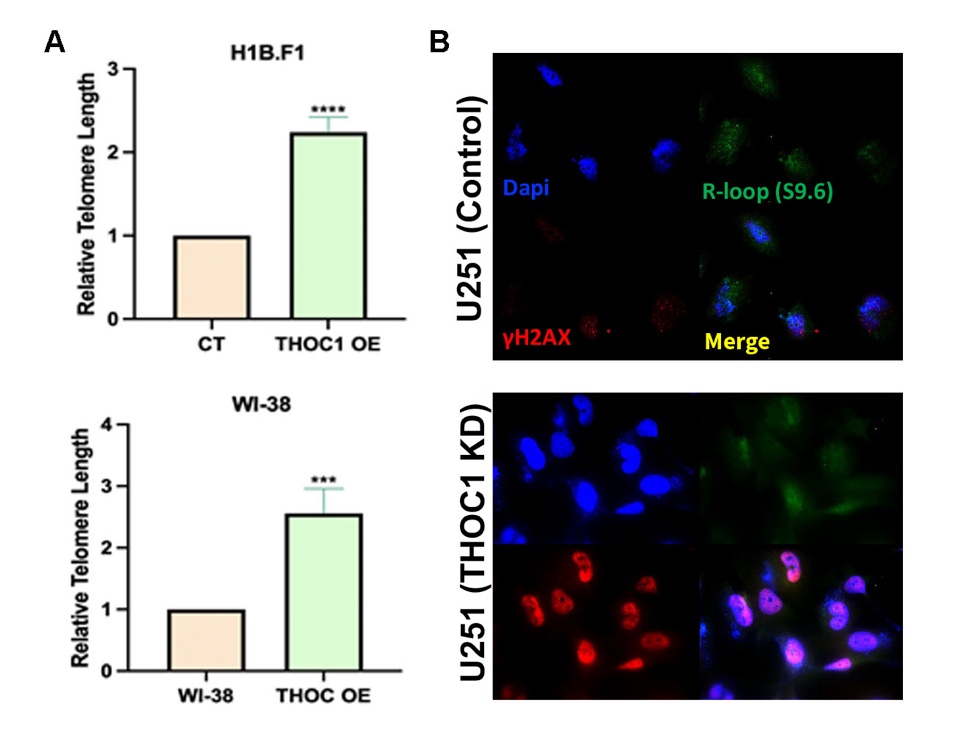


**Figure S6:** *THOC1 Overexpression Results in Increased Telomere Length.* A) THOC1 overexpression significantly increases telomere length in both H1B.F1 and WI-38 cells compared to controls. B) Immunohistochemical staining U251 control and THOC1 KD demonstrates increased R-loops and DNA damage marker γH2AX

**Supplementary Figure 7**


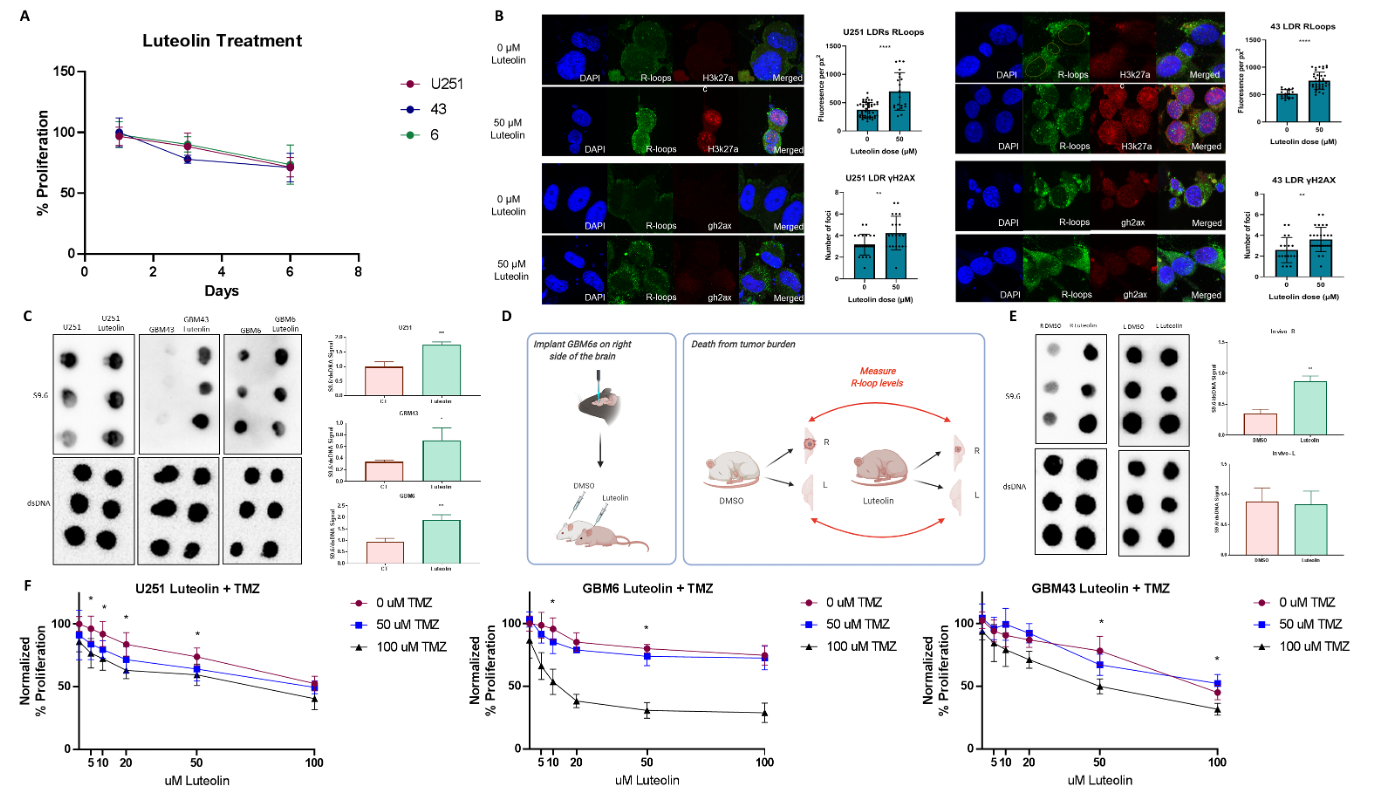


**Figure S7:** *Luteolin Targets THOC1 and Improves GBM Viability* A) MTT showing decreased proliferation of control and luteolin-treated U251, GBM43, and GBM6 cells over 6 days. B) Immunocytochemistry of R-loops and γH2AX in control and 50 uM luteolin-treated U251 and GBM43 cells, along with quantification analysis demonstrating higher levels of DNA damage. C) Dot blot analysis to assess R-loop levels, comparing control and 50 uM luteolin-treated U251, GBM43, and GBM6 cells, showing that luteolin treatment led to increased S9.6 **signal**. Nucleic acid extract of these lines were immunoblotted with antibody against S9.6 or an antibody against dsDNA as a control for equal loading. D) Schematic representing control and luteH1B.F1 cells intracranially implanted in mice. E) Dot blot analysis was performed in order to assess R-loop expression, comparing control and luteolin treated mice after intracranial implantation in both DMSO and luteolin-treated brain, demonstrating greater S9.6 signal in luteolin treated tumor tissue. F) Proliferation assays demonstrate additive effect of 0, 50, or 100 uM TMZ and increasing dosing of luteolin in U251, GBM6, and GBM43.
